# Identification of specific miRNAs in early-stage mung bean (*Vigna radiata*) using DNA/AgNCs sensors and miRNAtome analysis

**DOI:** 10.1093/hr/uhaf312

**Published:** 2025-11-13

**Authors:** Young Kyoung Oh, Hari Chandana Yadavalli, Christian Møller, Moon Young Ryu, Seok Keun Cho, Bora Lee, Mikyung Chang, Mi Young Byun, Jong Hum Kim, Hyun Ju Jung, Seong Wook Yang

**Affiliations:** Department of Systems Biology, Institute of Life Science and Biotechnology, Yonsei University, Seoul 03722, South Korea; Department of Systems Biology, Institute of Life Science and Biotechnology, Yonsei University, Seoul 03722, South Korea; Department of Systems Biology, Institute of Life Science and Biotechnology, Yonsei University, Seoul 03722, South Korea; Xenohelix Research Institute, BT Centre 305, 56 Songdogwahakro Yeonsugu, Incheon 21984, South Korea; Xenohelix Research Institute, BT Centre 305, 56 Songdogwahakro Yeonsugu, Incheon 21984, South Korea; Xenohelix Research Institute, BT Centre 305, 56 Songdogwahakro Yeonsugu, Incheon 21984, South Korea; Department of Systems Biology, Institute of Life Science and Biotechnology, Yonsei University, Seoul 03722, South Korea; Xenohelix Research Institute, BT Centre 305, 56 Songdogwahakro Yeonsugu, Incheon 21984, South Korea; Department of Life Science, Pohang University of Science and Technology (POSTECH), 77 Cheongam-Ro, Nam-Gu, Pohang, Gyeongbuk 37673, South Korea; Institute for Convergence Research and Education in Advanced Technology, Yonsei University, Seoul 03722, South Korea; Department of Systems Biology, Institute of Life Science and Biotechnology, Yonsei University, Seoul 03722, South Korea; Department of Systems Biology, Institute of Life Science and Biotechnology, Yonsei University, Seoul 03722, South Korea; Xenohelix Research Institute, BT Centre 305, 56 Songdogwahakro Yeonsugu, Incheon 21984, South Korea

## Abstract

MicroRNAs (miRNAs) are noncoding RNAs, ~21–24 nucleotides in length, that play a pivotal role in post-transcriptional gene regulation by inducing cleavage or translational repression of target mRNAs with complementary sequences. In this study, we identified miRNAs expressed during the early developmental stage of mung bean (*Vigna radiata*), a major legume crop, using small RNA sequencing (sRNA-seq), and analyzed their expression profiles across various mung bean tissues. Mung bean-specific miRNAs were found to be highly expressed in the aerial parts of seedlings, particularly in the leaves. Furthermore, the expression of these miRNAs was effectively validated using Tailed-Hoogsteen triplex DNA-encapsulated silver nanocluster (DNA/AgNC) sensors. The nanosensor enables rapid detection of target miRNAs within 30 min and is easy to apply for field-based assessments. The predicted target mRNAs of the identified miRNAs were associated with a range of biological processes relevant to early-stage development. This study highlights the potential of nanosensor-based approaches for the efficient identification of novel miRNAs in staple crops, offering a promising strategy to reduce the cost, time, and labor required during the transition from laboratory research to field applications.

## Introduction

Mung bean (*Vigna radiata*) is a nutritionally valuable crop widely cultivated in Asia and Africa due to its high content of protein, vitamins, and minerals. Its rich nutritional composition contributes significantly to food security and sustainable agriculture. In plant science, mung bean has been widely used as a model species for studying legume tissue development due to its short life cycle, well-defined differentiation stages, and high responsiveness to environmental cues. These characteristics make it suitable for investigating how external factors such as light, temperature, and water availability influence tissue growth and differentiation at the molecular level [1–4].

MicroRNAs (miRNAs) are small noncoding RNAs that play crucial roles in post-transcriptional gene regulation in plants [5–8]. Their biosynthesis and regulatory mechanisms have been extensively characterized [9]. MiRNAs are known to participate in various developmental processes and also modulate plant responses to environmental stimuli [6, 10–14]. In addition, species-specific miRNAs often regulate unique target genes associated with adaptation and developmental specificity [15]. Therefore, the identification of novel miRNAs in crops provides important insights into species-specific regulatory networks and offers valuable molecular resources for crop improvement [14, 16–18].

Recently, fluorescence-based nanosensor technologies, particularly DNA-encapsulated silver nanoclusters (DNA/AgNCs), have emerged as powerful tools for miRNA detection in both live cells and *in vitro* systems [19, 20]. These sensors can operate in ‘turn-off’ or ‘turn-on’ modes, depending on the probe design. For example, locking–unlocking hairpin DNA/AgNCs emit strong orange fluorescence upon recognition of specific miRNAs [21]. Similarly, Tailed-Hoogsteen triplex DNA/AgNCs exhibit fluorescence changes in response to pH, metal ions, or temperature and can act as sensitive turn-on sensors when equipped with a complementary tail sequence [22]. This system enables rapid detection within 30 min, requires only a simple two-step protocol, and provides a cost-effective platform for miRNA monitoring in agricultural research.

In this study, we performed small RNA sequencing (sRNA-seq) of mung bean seedlings, focusing on leaves, stems, and roots, to identify miRNAs involved in early growth and tissue differentiation. Selected miRNAs were further validated using the Tailed-Hoogsteen triplex DNA/AgNCs nanosensor, demonstrating its rapid and reliable performance and highlighting its potential for practical application in crop diagnostics.

## Results

### Small RNA profiling and differential expression of conserved and novel miRNAs during tissue development in mung bean

**Figure 1:**
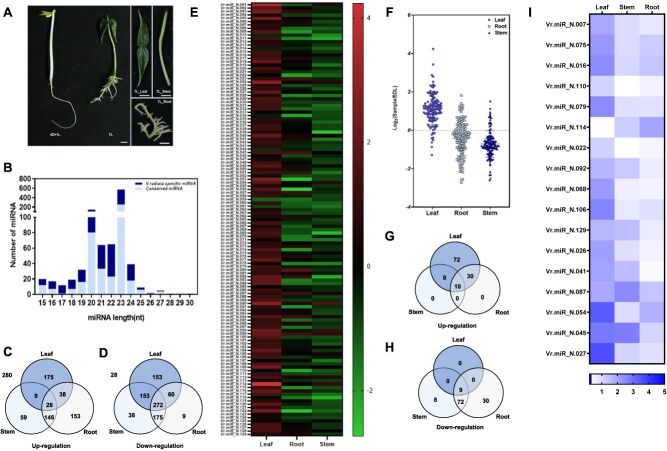
Profile of sRNA and novel mung bean miRNAs in mung bean. sRNA profiles in mung bean were generated by sRNA sequencing. (A) Representative image of mung bean seedlings used for sRNA sequencing (4D + 1L: mung bean seedlings grown for 4 days at dark conditions followed by 1 day of incubation at light conditions; 7L: mung bean seedlings grown for 7 days at light conditions. Leaf, stem, and root tissues from 7L seedlings were harvested separately for analysis. (B) Size distribution of sRNAs showing the proportion of conserved and nonconserved miRNA in *Viridiplantae.* (C, D) Venn diagrams illustrating differentially expressed sRNAs based on normalized sequencing data, showing (C) upregulated and (D) downregulated sRNAs in mung bean. Numbers outside the Venn diagrams indicate sRNAs not included in either category. (E, F) Expression profiles of all novel mung bean miRNAs identified by sequencing. (G, H) Venn diagrams showing (G) upregulated and (H) downregulated novel mung bean miRNAs based on normalized expression levels. (I) Expression profile of 17 high-confidence novel mung bean miRNAs across different tissues.

Tissue development is a complex process regulated by various sRNAs, including miRNAs, which play critical roles in gene expression [6, 8, 23, 24]. To investigate the role of miRNAs during early development in mung bean, we performed sRNA profiling using tissue samples from 7-day-old seedlings and compared them with those from 5-day-old seedlings (Fig. 1A). To examine the differential expression of conserved and novel miRNAs, total miRNA read counts from leaf, stem, and root tissues were normalized to those of the 4D + 1L control (seedlings grown in the dark for 4 days, followed by 1 day in light). In total, 1016 predicted miRNAs were identified in the 7-day-old light-grown seedlings through sRNA-seq. Among these, 470 miRNAs shared conserved sequences with *Arabidopsis* or other *Viridiplantae*, while 546 miRNAs were mung bean-specific (Fig. 1B). The majority of the identified miRNAs ranged from 20 to 24 nucleotides, and distinct size distributions were observed across the four sample types analyzed: 4D + 1L, leaf, stem, and root (Fig. S1A). Of the 1016 miRNAs identified (normalized expression in TPTM), 250 miRNAs were upregulated in leaf, 242 in stem, and 365 in root tissues (Fig. 1C). Notably, 28 miRNAs were consistently upregulated across all three tissues. In contrast, 638 miRNAs were downregulated in leaf and stem, and 516 were downregulated in root (Fig. 1D). These results indicate that both conserved and novel miRNAs exhibit strong tissue-specific expression patterns and likely contribute to the regulation of growth and development in mung bean. Previously, we identified a mechanism termed ‘miRNA-biogenetic inconsistency’, which explains the reduction in miRNA levels in dark-grown seedlings upon exposure to light [25]. We further demonstrated that this process is regulated by ‘FHA2’, a negative regulator of miRNA biogenesis [26]. Consistent with this, we observed a similar reduction in overall miRNA levels in light-treated mung bean seedlings, suggesting that miRNA biogenesis is finely regulated during photomorphogenesis. To further characterize the novel mung bean-specific miRNAs, we analyzed their tissue-specific expression profiles. Most of the novel miRNAs were upregulated in leaf samples but downregulated in root and stem tissues (Fig. 1E and F). Specifically, 120 novel miRNAs were upregulated in leaves, including 72 that were leaf-specific (Fig. 1G). In comparison, 18 and 40 miRNAs were upregulated in the stem and root, respectively, and only 10 novel miRNAs were consistently upregulated across all three tissues. Conversely, 119 novel miRNAs were downregulated overall, with only nine showing consistent downregulation across all tissues. Stem and root tissues contained 89 and 111 downregulated novel miRNAs, respectively (Fig. 1H). The 25 most highly expressed novel miRNAs were visualized with their expression patterns using a heatmap (Fig. S1B; Table S4). To enhance the accuracy of novel miRNA candidate selection, we applied a more stringent screening process incorporating primary miRNA secondary structure analysis and target gene-binding energy evaluation (Fig. S1C). RNAhybrid analysis was conducted to assess the binding stability between the novel miRNAs and their predicted targets. As a result, 17 mung bean-specific miRNAs were stringently selected (Fig. 1I). All 17 miRNAs exhibited strong binding potential, with minimum free energy (MFE) values ranging from −31.2 to −45.7 kcal/mol and minimum duplex energy (MDE) values from −34.7 to −47.5 kcal/mol. Because binding energies below −20 kcal/mol are generally considered biologically meaningful, these data suggest that the identified miRNAs are predicted to form stable miRNA–mRNA interactions and may effectively regulate their target genes (Fig. S1D). The most strongly upregulated miRNAs were predominantly expressed in leaf tissues.

### Establishment of Tailed-Hoogsteen triplex DNA/AgNCs to detect mung bean miRNAs

**Figure 2:**
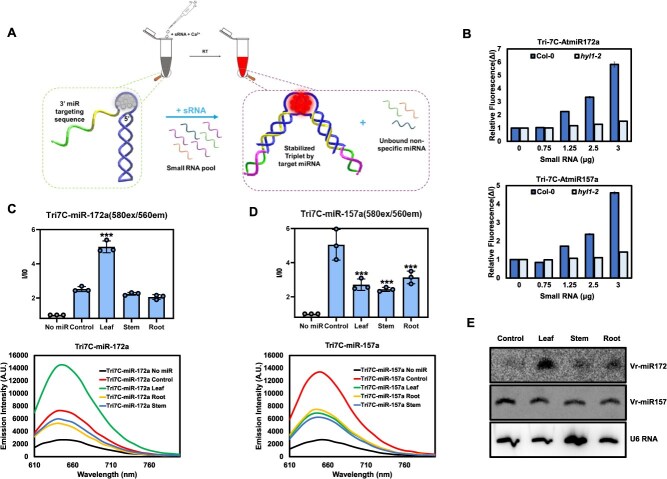
*In vivo* confirmation of mung bean novel miRNA levels using Tri-7C DNA/AgNCs. Validation of Tri-7C DNA/AgNCs for miRNA detection. (A) Schematic representation of Tri-7C DNA/AgNCs mechanism in the presence and absence of sRNA, (B) Concentration-dependent detection of miRNAs using Arabidopsis sRNAs. (C–E) Detection of conserved miRNAs from mung bean using (C, D) Tri7C DNA/AgNCs sensors and (E) northern blot analysis. Emission signals of Tri-7C/AgNCs were measured from three independent replicates and are presented as mean ± SD. Statistical significance was determined using a two-tailed unpaired Student’s *t*-test. *P* < 0.05 was considered statistically significant. Asterisks indicate significant differences compared to the control condition: *P* < 0.05 (^*^), *P* < 0.01 (^**^), *P* < 0.001 (^***^).

Following miRNA profiling by next-generation sequencing (NGS), the expression levels of specific miRNAs are typically validated using sRNA blot analysis or quantitative reverse transcription-polymerase chain reaction (qRT-PCR)-based methods. However, these approaches are time-consuming—often requiring several days—and are limited to controlled laboratory settings. To overcome these limitation, we developed point-of-care testing methods for monitoring miRNAs and siRNAs that can be applied in agricultural practices [22]. One such approach is the ‘Tailed-Hoogsteen triplex DNA**-**encapsulated silver nanocluster (DNA/AgNC) sensor’, which enables miRNA detection in both animal and plant systems within 30 min [22]. The sensor is based on the Tri7C DNA structure, extended with a 3′-tail complementary to the target miRNA. Upon hybridization, it forms a rigid RNA–DNA A-form duplex, thereby stabilizing the Hoogsteen triplex structure. This stabilized conformation enhances silver nanocluster fluorescence, producing a detectable red emission signal indicative of the presence of target miRNA [22] (Fig. 2A). HYL1 is a key RNA-binding protein that is essential for miRNA biogenesis in plants. In its absence, most miRNAs are maintained at minimal levels. To evaluate the performance of the nanosensor system, we initially compared the detection of miR172a and miR157a in *hyl1-2* mutant and wild-type (WT) *Arabidopsis thaliana* seedlings. sRNAs were extracted from both *hyl1-2* and WT plants and incubated with the Tri7C-AtmiR-172a and Tri7C-AtmiR-157a sensors for 30 min. As shown in Fig. 2B, the red fluorescence intensity of both sensors was markedly reduced in the *hyl1-2* mutant compared to WT, consistent with the established role of HYL1 in miRNA biogenesis. We next applied the Tri7C-VrmiR-172a and Tri7C-VrmiR-157a sensors to sRNAs extracted from mung bean seedlings (4D + 1L), including samples from leaf, stem, and root tissues. Both Vr-miR172a and Vr-miR157a were successfully detected across tissues, with Vr-miR172a showing notably higher expression in leaf tissues (Fig. 2C and D). To confirm the accuracy of the nanosensor detection, we performed sRNA blot analysis, which showed strong concordance with the sensor results (Fig. 2E). Collectively, these findings indicate that the Tailed-Hoogsteen triplex DNA/AgNC sensor provides precise, rapid, and reliable miRNA detection, highlighting its potential applicability in crop research and field-based applications.

### Detection of mung bean-specific miRNAs using Tailed-Hoogsteen triplex DNA/AgNCs

**Figure 3:**
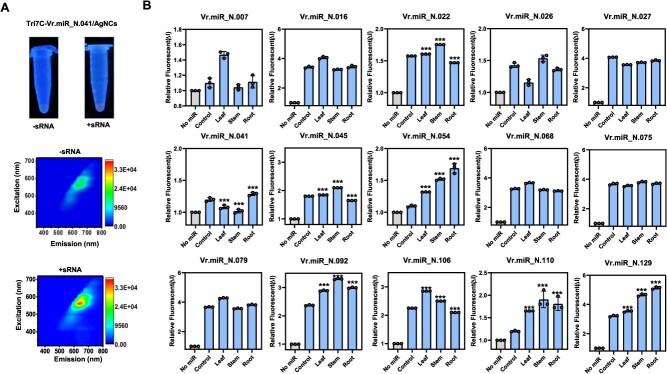
*In vivo* confirmation of mung bean novel miRNA levels using Tri-7C DNA/AgNCs. Confirmation of mung bean novel miRNA using Tri-7C DNA/AgNCs nanosensors. (A) Representative pictures and full-scan plots showing detection of novel miRNA Vr.miR_N.041 (B) Detection of 15 novel mung bean miRNAs using Tri-7C DNA/AgNCs sensors. Fluorescence spectra were recorded at 560 nm excitation and 600–700 nm emission spectrum. The relative fluorescence intensity reflects the miRNA quantity across mung bean sRNA samples. Emission signals were measured from three independent replicates and are presented as mean ± SD. Statistical significance was determined using a two-tailed unpaired Student’s *t*-test. *P* < 0.05 was considered statistically significant. Asterisks indicate significant differences compared to the control condition: *P* < 0.05 (^*^), *P* < 0.01 (^**^), *P* < 0.001 (^***^).

Next, to monitor newly identified miRNAs in mung bean, we designed Tri7C-VrmiR sensors for eight selected miRNAs, chosen for their high predicted interaction scores (S) with target mRNAs. These custom-designed nanosensors were incubated with purified sRNAs extracted from the leaf, stem, and root tissues of 7-day-old seedlings. Sensor fluorescence was measured at excitation/emission wavelengths of 580/650 nm. The Tri7C-VrmiR_N.041 sensor showed ~1.5-fold increase in red fluorescence when mixed with sRNAs from leaf tissue, confirming its responsiveness to the target miRNA (Fig. 3A). Subsequently, we measured the fluorescence intensity of all 15 designed Tri7C-VrmiR sensors, all of which displayed distinct tissue-specific fluorescence patterns. For example, Vr.miR_N.022, Vr.miR_N.045, Vr.miR_N.092, and Vr.miR_N.110 exhibited markedly increased fluorescence in stem tissue, whereas Vr.miR_N.129 showed the highest intensity in root tissue. In contrast, Vr.miR_N.007, Vr.miR_N.041, Vr.miR_N.054, and Vr.miR_N.106 showed strong fluorescence signals in leaf tissue (Fig. 3 and Fig. S2A). Contour plots of excitation versus emission spectra confirmed the effectiveness of each Tri7C-VrmiR sensor as mung bean-specific miRNA sensors. The addition of mung bean sRNAs enhanced red fluorescence across all sensors, with a characteristic signal at 580/650 nm. In contrast, two human-specific sensors, Tri7C-HsmiR21 and Tri7C-HsmiR9, used as negative controls, showed no fluorescence signal when mixed with mung bean sRNAs (Fig. S2B). These results support the specificity and applicability of the nanosensor system for rapid, tissue-specific monitoring of mung bean miRNAs. To investigate whether the 15 selected miRNAs were processed from authentic precursors, we analyzed the predicted secondary structures of their corresponding pri-miRNAs. All were found to be consistent with canonical pri-miRNA stem-loop structures (Fig. S3). Based on their distinct tissue-specific fluorescence patterns, we selected eight representative miRNAs for further validation by qRT-PCR. We then assessed the expression levels of their corresponding pri-miRNAs using qRT-PCR (Fig. S4). Pri-miRNA levels are typically inversely correlated with the abundance of mature miRNAs. For example, when miRNA processing is impaired or slowed, mature miRNA levels decrease while pri-miRNA transcripts accumulate. Conversely, enhanced processing efficiency typically leads to an increase in mature miRNA levels and a corresponding decrease in pri-miRNA abundance [25, 27, 28]. However, this inverse relationship can be influenced by additional factors. The promoter activity of *MIRNA* genes may directly affect pri-miRNA transcript levels, and the stability or half-life of mature miRNAs may vary in a tissue-specific manner. Considering these variables, we reasoned that the expression levels of the tested pri-miRNAs did not show a clear inverse correlation with their corresponding mature miRNAs. Taken together, these findings suggest that the newly identified mung bean miRNAs are expressed in specific tissues and may regulate genes associated with developmental processes (Fig. 1I).

### Gene ontology analysis of mung bean miRNA target genes

To explore the biological roles of mung bean-specific miRNAs, we analyzed their predicted target genes and performed Gene Ontology (GO) and Kyoto Encyclopedia of Genes and Genomes Pathway (KEGG pathway) analyses using the DAVID tool [29]. The target genes were associated with a variety of cellular components, including mitochondria, nucleus, plasma membrane, endoplasmic reticulum (ER), and chloroplast (Fig. S5A and B). In terms of molecular function, many target genes displayed RNA-binding and DNA-binding activities, and several are involved in enzymatic functions related to diverse metabolic pathways (Fig. S5C). With respect to biological processes, the predicted targets of the novel miRNAs were implicated in a wide range of functions, including hormone signal transduction, transport activity, transcriptional regulation, pollen and embryo development, DNA repair, RNA processing, root development, and various catabolic processes (Fig. S5D). Collectively, these findings suggest that the identified novel miRNAs may regulate target genes involved in early-stage growth and development in mung bean.

### Differential expression of miRNA target genes during early growth of mung bean

**Figure 4:**
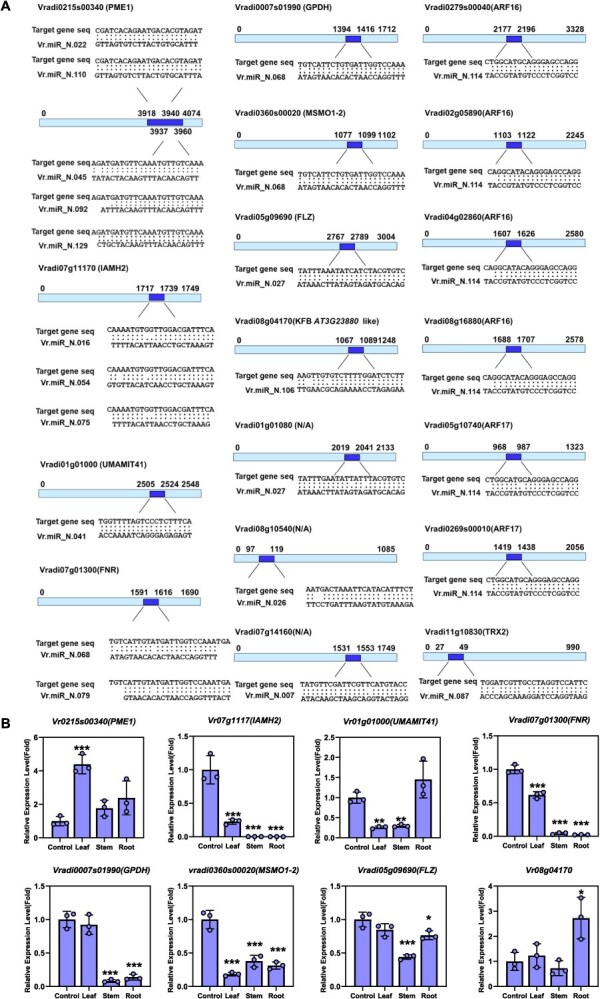
Transcript level of novel mung bean miRNA-targeted genes in tissues. Expression patterns of 15 predicted target genes of novel mung bean miRNAs validated by qRT-PCR. (A) Schematic representation of novel mung bean miRNAs and their corresponding target gene sequence. (B) Relative transcript abundance was measured from control (4D + 1L), leaf, stem, and root samples of mung bean, with data normalized to internal reference genes (*EF1a*), highlighting differential expression linked to miRNA targeting. The data were analyzed using *n* = 3 biological replicates. Statistical significance was determined using a two-tailed unpaired Student’s *t*-test. *P* < 0.05 was considered statistically significant. Asterisks indicate significant differences compared to the control condition: *P* < 0.05 (^*^), *P* < 0.01 (^**^), *P* < 0.001 (^***^).

Based on *in silico* miRNA analysis, we validated the expression levels of their predicted target genes in mung bean tissues. First, we identified target genes containing complementary sequences to the selected miRNAs (Fig. 4A). miRNA target pairs with uncharacterized target genes (*Vradi01g01080, Vradi07g14160,* and *Vradi08g10540*) or undetectable mature miRNAs (Vr.miR_N.087 and Vr.miR_N.114) were excluded from further analysis. qRT-PCR was performed to examine the expression of target genes with high expectation scores for miRNA–mRNA pairing. Total RNA was extracted from 4D + 1L seedlings (control), as well as from leaf, stem, and root tissues of 7-day-old light-grown seedlings. Among the confirmed mung bean-specific miRNAs, Vr.miR_N.022, Vr.miR_N.045, Vr.miR_N.092, Vr.miR_N.110, and Vr.miR_N.129 were all predicted to target the same gene, *Vradi0215s00340*, which encodes Pectin Methylesterase 1 (PME1). PME1 is an enzyme that mediates pectin modification during cell wall remodeling and plays a crucial role in cell expansion, thereby influencing the growth of roots, stems, and leaves [30]. Interestingly, Vr.miR_N.022, Vr.miR_N.045, Vr.miR_N.092, and Vr.miR_N.110 showed peak expression in stem tissue, where *PME1* expression was notably lower compared to leaf and root, suggesting a possible miRNA–mRNA pairing (Fig. 4B). In contrast, Vr.miR_N.129 exhibited its highest expression in leaf tissue, where *PME1* expression was also elevated (Figs 3B and 4B). These data indicate that the combined expression levels of these miRNAs may predominantly influence the degree of *PME1* repression. *Vradi07g111710*, the predicted target of Vr.miR_N.016, Vr.miR_N.054, and Vr.miR_N.075, shows high-sequence homology to *Arabidopsis indole-3-acetamide hydrolase* (*IAMH2*), which is involved in auxin biosynthesis and contributes to multiple developmental process [31]. Vr.miR_N.054 expression peaked in root tissue but remained ~130%–170% higher than the control across all tissues. Although Vr.miR_N.016 and Vr.miR_N.075 are also predicted to target *IAMH2*, *IAMH2* did not exhibit tissue-specific expression, suggesting a limited regulatory role. Consistently, *VrIAMH2* expression was strongly reduced in all tissues compared to the control, indicating that Vr.miR_N.054 may predominantly repress *VrIAMH2* expression during early mung bean development (Fig. 4B). Vr.miR_N.041 targets *Vradi01g1000*, a homolog of *Arabidopsis UMAMIT41*, which encodes a member of the USUALLY MULTIPLE ACIDS MOVE IN AND OUT TRANSPORTERS family. Vr.miR_N.041 showed high expression in control and root tissues, but was reduced in leaf and stem. Its predicted target gene, *Vradi01g1000*, was also markedly reduced in leaf and stem tissues. This apparent inconsistency suggests two possibilities: either Vr.miR_N.041 and *Vradi01g1000* may not form a true miRNA–mRNA regulatory pair, or the reduced expression of *Vradi01g1000* in leaf and stem may be due to transcriptional repression or low promoter activity in those tissues (Fig. 4B). *Vradi0007s01990,* encoding Glycerol-3-phosphate dehydrogenase (GPDH), a key enzyme in glycerolipid biosynthesis, was predicted to be targeted by Vr.miR_N.068. Although Vr.miR_N.068 expression remained largely unchanged in leaf, *VrGPDH* expression was significantly reduced in stem and root tissues (Fig. 4B). Another predicted target of Vr.miR_N.068 is *Vradi0360s00020*, a homolog of *methylsterol monooxygenase 1-2* (*MSMO1-2*), which functions in sterol biosynthesis and may affect auxin distribution by altering membrane composition and fluidity. Despite the absence of a clear tissue-specific pattern in Vr.miR_N.068 expression, *VrMSMO1-2* expression was notably reduced across all tested tissues (Fig. 4B). These findings suggest a potential regulatory relationship, though definitive confirmation would require appropriate negative controls. Similarly, *Vradi07g01300,* encoding a *Ferredoxin-NADP^+^ reductase* (*FNR*)*,* was identified as a potential target of Vr.miR_N.068 and N.079. *FNR* is essential for photosynthetic NADPH generation and also functions in nonphotosynthetic plastids during early development, maintaining reactive oxygen species (ROS) homeostasis and cellular redox balance. Neither Vr.miR_N.068 nor Vr.miR_N.079 showed tissue-specific expression (Fig. 3B). Nonetheless, *VrFNR* expression was significantly reduced across all tissues, particularly in stem and root, suggesting the involvement of additional, uncharacterized regulators (Fig. 4B). *Vradi05g09690* encodes an FCS-like Zinc Finger (FLZ) protein involved in energy signaling via the SnRK1 complex, which was predicted to be regulated by Vr.miR_N.027. Expression of Vr.miR_N.027 remained largely unchanged across tissues, with only minor variation in stem. A similar trend was observed for *VrFLZ* expression, suggesting that Vr.miR_N.027 may exert a modest, tissue-dependent regulatory influence (Fig. 4B). Vr.miR_N.106 targets *Vradi08g04170*, a gene homologous to F-box/kelch-repeat protein-*AT3G23880-*like in *Arabidopsis*. Both Vr.miR_N.106 and its target gene exhibited relatively stable expression across most tissues, except for a distinct change in the root. This pattern suggests that Vr.miR_N.106 may play a limited, possibly root-specific regulatory role (Fig. 4B).

In several cases, elevated miRNA expression corresponded with reduced target gene expression, supporting a potential regulatory interaction. In others cases, target repression occurred without a corresponding change in miRNA abundance, implying the involvement of additional post-transcriptional or transcriptional mechanisms. Overall, nanosensor-based measurements of selected miRNAs were largely consistent with the expression patterns of their predicted targets, supporting an inverse correlation between miRNAs and target mRNAs.

### Validation of mung bean miRNA processing

**Figure 5:**
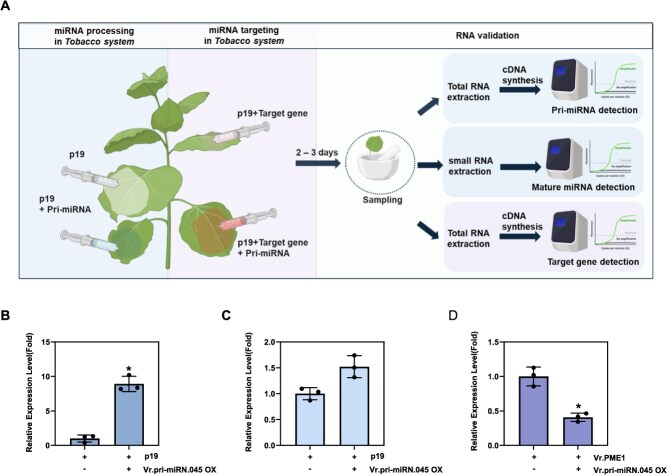
Validation of novel mung bean miRNA processing in plant and regulatory pathway of mung bean miRNAs and their target genes. Validation of mung bean novel pri-miRNAs processing and its regulatory relationship with target genes. (A) Schematic representation of the transient expression assay in tobacco showing miRNA processing and target validation workflow. For miRNA processing validation, pri-miRNA constructs were co-infiltrated with p19, and both pri-miRNA (first section of RNA validation) and mature miRNA (second section of RNA validation) levels were analyzed by qPCR. The p19-only condition served as a negative control. For miRNA targeting validation, pri-miRNA and target gene constructs were co-infiltrated with p19, and target gene expression levels were analyzed by qPCR (last section of RNA validation). The p19 with target gene condition served as a control. Relative expression level of (B) pri-miRNA and (C) mature miRNA in tobacco leaves. (D) Relative target gene (*Vr.PME1*) expression level in cotransformation of miRNA and its targets in tobacco leaves. Figure 5A was created in BioRender. Statistical significance was determined using a two-tailed unpaired Student’s *t*-test. *P* < 0.05 was considered statistically significant. Asterisks indicate significant differences compared to the control condition: *P* < 0.05 (^*^), *P* < 0.01 (^**^), *P* < 0.001 (^***^).

To verify whether mung bean-specific miRNAs are actively processed in plants, we conducted a miRNA–mRNA silencing assay in *Nicotiana benthamiana* leaves to assess the processing of mung bean pri-miRNAs. Because generating transgenic mung bean lines or performing transient assays directly in mung bean is technically challenging, we utilized the tobacco system as a heterologous platform. This system allows efficient transient expression and simultaneous delivery of multiple gene constructs, making it well suited for evaluating miRNA–target interactions (Fig. 5A). One of the identified mung bean pri-miRNAs, Vr.miR_N.045, was transiently overexpressed via agroinfiltration. Three days postinfiltration, pri-miR_N.045 showed strong expression in tobacco leaves (Fig. 5B). The presence of the processed, mature Vr.miR_N.045 was further confirmed by qRT-PCR (Fig. 5C), indicating that the mung bean pri-miRNA was properly processed into its mature form in the tobacco system. To assess the regulatory activity of the processed miRNA, we co-expressed the target gene *PME1* together with Vr.miR_N.045. The transcript level of *PME1* was reduced compared to samples without pri-miRNA co-expression (Fig. 5D), demonstrating the gene-silencing effect of Vr.miR_N.045. Taken together with the miRNA expression analyses and GO, these findings suggest that a substantial subset of mung bean-specific miRNAs are actively processed and functionally engaged in regulating key genes associated with leaf and root growth during early seedling development.

## Discussion

miRNAs play critical roles in regulating plant development, stress responses, and disease resistance. Identification of novel, species-specific miRNAs in crops is essential for advancing our understanding of their unique regulatory functions [16, 32]. In this study, we identified 129 novel, tissue-specific miRNAs expressed during early-stage mung bean seedlings, aiming to elucidate their roles in the growth and development of this important legume crop. Using sRNA-seq and a novel detection method—Tailed-Hoogsteen DNA/AgNCs sensors—we validated the expression of these mung bean-specific miRNAs. Our analyses revealed that many of these miRNAs were highly expressed in light-grown seedlings, particularly in leaf tissue, suggesting the involvement of a complex regulatory network underlying leaf development. We further confirmed that the predicted target genes of these miRNAs are involved in critical developmental pathways. Specifically, several target genes participate in auxin signaling, a key hormonal pathway that governs cell division, expansion, and differentiation during early growth stages [30, 31, 33](Fig. S5). Notably, the target genes *PME1, GPDH,* and *MSMO1-2* are associated with cell expansion and are likely to influence early plant growth. In addition, *FNR* is involved in photosynthesis, while *IMAH2* participates in the biosynthesis of auxin, a key plant growth hormone. Comparable to *A. thaliana*, where miRNAs such as miR319 and miR396 regulate leaf morphogenesis and proliferation [34, 35], our findings suggest that miRNAs in mung bean serve a similarly central role in balancing cell growth and expansion during early development [6, 13, 36]. During early mung bean growth, both target genes and their corresponding miRNAs showed an overall increase in expression. This suggests that mung bean-specific miRNAs may fine-tune gene expression levels essential for rapid organogenesis. Consistent with recent reports, not all miRNA–target gene pairs exhibited strict inverse expression pattern between miRNA and target transcript levels. In our dataset, five out of eight predicted target genes did not display a clear inverse relationship with their cognate miRNAs, suggesting that miRNA–mRNA regulation may be modulated by complex regulatory mechanisms. For example, endogenous target mimics can inhibit miRNA function; in plants, *INDUCED BY PHOSPHATE STARVATION 1* (*IPS1)* sequesters miR399 to protect *PHOSPHATE 2 (PHO2)* transcripts, while in animals, it is well known that lncRNAs and circRNAs act as sponges to bind and suppress miRNAs [37]. Translational repression, rather than mRNA cleavage, also influences the inverse correlation between miRNAs and their target mRNAs, although it primarily reduces protein levels [38]. Together, these factors could explain why some predicted targets are not downregulated despite the presence of their cognate miRNAs.

Previous research in *Vigna mungo* (black gram) has shown that miRNAs such as miR156, miR159, and miR398 participate in immune signaling via targets such as nucleotide-binding leucine-rich repeat proteins (NB-LRR), MYB transcription factors, and superoxide dismutase (SOD) [39]. Recent studies in legumes such as soybean, pigeon pea, and common bean have revealed that the miRNA repertoire includes both highly conserved families and species-specific variants [40–45]. While conserved miRNAs such as miR156 and miR167 regulate fundamental developmental processes across species, species-specific miRNAs are thought to contribute to adaptive diversification. In our study, we identified several novel miRNAs specifically expressed during early mung bean seedling development, which are absent from existing legume miRNA databases. These miRNAs may represent mung bean–specific regulators that emerged through recent evolutionary divergence, contributing to the diversification of developmental regulation in this species.

In this study, we employed a nanosensor system to validate novel miRNAs in mung bean, demonstrating its potential to complement conventional miRNA discovery pipelines by providing an additional layer of biological validation. Nevertheless, high GC content in target genes continues to pose experimental challenges for probe design and nanosensor application. However, by directly using total RNA extracted from plant tissues, this approach bypasses the need for reverse transcription and other preprocessing steps, significantly simplifying the workflow. Additionally, it enables the sensitive and accurate detection of sRNA expression levels, outperforming traditional methods. This capability is particularly valuable for nonmodel crops with limited genomic annotation, where distinguishing biologically relevant miRNAs from background noise remains a major challenge. Our findings also underscore the applicability of this nanotechnology-based detection platform as a field-deployable system for *in planta* monitoring of sRNAs. While previous studies have demonstrated its functionality in animal systems, our study provides the first evidence of its effectiveness in crop species. Compared with PCR-based and other instrument-intensive methods requiring sophisticated instrumentation, our nanosensor offers a simple, portable, and rapid alternative for direct quantification of sRNAs from plant samples. This makes it highly suited for on-site applications in agricultural settings.

In summary, this work expands the catalog of species-specific miRNAs in mung bean and identifies their potential target genes in early plant development. Moreover, the Hoogsteen triplex DNA/AgNCs nanosensor represents a promising tool for on-site detection of plant miRNAs and siRNAs, with broad potential for agricultural diagnostics and crop stress monitoring.

## Materials and methods

### Plant preparation

Mung bean (*Vigna radiata*) seeds were sterilized with 20% bleach for 3 min, repeated three times, and then rinsed 10 times with sterilized water. After sterilization, seeds were incubated at 37°C for 2 h with shaking to break dormancy and promote germination, followed by a brief rinse with sterilized water. The seeds were sown on half-strength Murashige and Skoog (MS) medium and grown at 23°C under constant light for 5–7 days. At 7 days after germination, whole seedlings or individual tissues (leaf, stem, or root) were collected and immediately frozen in liquid nitrogen. Leaf tissue was sampled from the region above the apical hook, stem tissue from the segment between the apical hook and the seed, and root tissue from the portions below the seed. As a control, sterilized seeds were germinated in complete darkness for 4 days and subsequently exposed to light for 1 day (4D + 1L).

*Arabidopsis thaliana* WT Col-0 and *hyl1-2* mutant seeds (Stock No. CS6602) were obtained from the Arabidopsis Biological Resource Center (ABRC). Seeds were sterilized with 20% bleach for 10 min and rinsed 10 times with sterilized water. Following sterilization, seeds were sown on half-strength MS medium and grown at 23°C under constant white light for 10 days [46]. The resulting seedlings were harvested and immediately frozen in liquid nitrogen.

### Small RNA purification for miRNA detection

sRNAs were extracted from the harvested mung bean tissues using the XENOPURE sRNA purification kit (Xenohelix, South Korea). The samples were ground in liquid nitrogen, followed by RNA precipitation according to the manufacturer’s protocol. The resulting sRNA pellets were dissolved in RNase-free water, and RNA concentration was measured using Nanodrop One spectrophotometer (Thermo Fisher, United States). The purified sRNAs were subsequently used for miRNA detection via the DNA/AgNCs nanosensor monitoring system and northern blot analysis.

### Small RNA sequencing and data analysis

For sRNA-seq, total sRNA was extracted from mung bean samples as described above using XENOPURE small RNA purification kit (Xenohelix, South Korea). sRNA-seq and library construction were outsourced (LAS and Decode cell, South Korea) using Illumina platform. Raw reads were subjected to quality control, adapter trimming, and collapsing of identical reads into unique sequences. The processed reads were mapped to the mung bean reference genome, and both known and novel miRNAs were predicted using the miRDeep2 pipeline [47]. Hairpin structures were evaluated with the Vienna RNA package. MiRNA expression levels were quantified and normalized as transcripts per 10 million (TPTM), calculated by dividing raw miRNA counts by the total number of clean reads in each sample. Differential expression analysis of miRNAs was conducted across three tissues (leaf, stem, and root) in comparison with 4D + 1L seedlings, as described above. Each sample was represented by a double biological replicate.

### Oligonucleotides and chemicals for Tailed-Hoogsteen triplex DNA-encapsulated silver nanocluster detection

All oligos were obtained from Bionics and IDT (South Korea). Diethyl pyrocarbonate (DEPC)-treated water was used to dissolve the oligos to a stock concentration of 100 μM. Sodium borohydride (NaBH_4_, 99.99%), silver nitrate (AgNO_3_, >99.99%), TRIZMA acetate salt (≥99.0%, from Sigma–Aldrich), magnesium nitrate (Mg(NO_3_)_2_, 99%), and 100% isopropanol were sourced from Sigma–Aldrich (USA). All salts and buffers were dissolved in Milli-Q water (18.2 MΩ cm). Calcium nitrate tetrahydrate (Ca(NO_3_)_2_·4H_2_O, 97%), and magnesium nitrate hexahydrate (Mg(NO_3_)_2_·6H_2_O, 98%) were purchased from Daejung Chemicals (South Korea).

### Synthesis of DNA/AgNCs for miRNA detection

For the synthesis of DNA/AgNCs, a reaction mixture containing Tri7C–miR probes (15 μM DNA oligo, Table S1), 4 mM Mg (NO_3_)_2_, and 10 mM phosphate buffer (pH 6.5) was prepared in a total volume of 25 μl. Samples were denatured at 95°C for 10 min and annealed at 25°C for 20 min. DNA/AgNCs were synthesized by adding AgNO_3_ and NaBH_4_ in a 1:1 ratio to a final concentration of 250 μM in a total reaction volume of 50 μl. After incubating at 25°C for 40 min, 40 mM of Ca(NO_3_)_2_ was added.

To detect the expression levels of novel mung bean miRNAs, 0.5 μg of purified sRNA was added to the respective Tri7C-miR DNA/AgNCs and incubated at 25°C for 20 min. For sensor-only samples, an equal volume of DEPC-treated water was added. All samples were diluted to a final volume of 200 μl prior to fluorescence measurement. Fluorescence spectra were measured using a 96-well plate reader (CLARIOstar, BMG Labtech).

### Northern blot

For the northern blotting, sRNAs were extracted from mung bean samples as described above using the XENOPURE Small RNA Purification Kit (Xenohelix, South Korea) and dissolved in 50% formamide. A total of 10 μg of sRNA was resolved using a 13% denaturing polyacrylamide gel and transferred onto a nylon membrane (Amersham, UK). Hybridization was performed with 5′-end-labeled DNA probes for 24 h, followed by two washes with washing buffer (2 × SSC, 0.1% SDS) for 10 min each. miRNA signals were detected using a Typhoon phosphor-imager scanner (Amersham, UK).

### Investigation of pri-miRNA sequences and prediction of their hairpin structures

Pri-miRNA sequences were retrieved from *V. radiata* genome data (Ensembl Plants; https://plants.ensembl.org/Vigna_radiata). Sequences were cross-checked using the NCBI Genome Data Viewer to exclude coding gene regions. RNA hairpin structures were predicted using the RNAfold Webserver with default parameters (http://rna.tbi.univie.ac.at/cgi-bin/RNAWebSuite/RNAfold.cgi).

### Target gene prediction

Identified novel mung bean miRNAs were used for target prediction based on the *V. radiata* genome assembly from Ensembl Plants (https://plants.ensembl.org/Vigna_radiata). Target genes were predicted using the psRNATarget online server with default parameters (https://www.zhaolab.org/psRNATarget). MFE and MDE values were calculated using RNAhybrid [48]. Targets with MFE < −20 kcal/mol were considered significant.

### Gene ontology analysis

Predicted target genes were annotated using DAVID web server (https://david.ncifcrf.gov/home.jsp) with ENSEMBLE_GENE_ID as the identifier [29, 49]. GO classification and pathway enrichment were conducted based on KEGG pathway.

### Quantitative reverse transcription PCR analysis

Samples were ground in liquid nitrogen, and total RNA was extracted using the XENOPURE Total RNA Purification kit (Xenohelix, South Korea). One microgram of RNA was used for cDNA synthesis using Xeno cDNA Synthesis kit (Xenohelix, South Korea). qRT-PCR was conducted using Xeno Green Premix (Xenohelix, South Korea) on a real-time thermal cycler (Takara, Japan). Primers were designed using the Primer3 web server (https://www.primer3plus.com/index.html), and sequences are listed in Tables S2 and S3. Relative expression levels were normalized to *EF1a* [50]. Three biological replicates were analyzed, and fold changes were calculated using the 2^−ΔΔCT^ analysis method.

### MiRNA processing experiments in tobacco leaves

Predicted pri-miRNA sequences from mung bean were cloned into the pEarleyGate vector and transformed into *Agrobacterium tumefaciens* (GV3101 strain). For transient expression, Agrobacterium cultures were suspended in infiltration buffer (10 mM MES, 10 mM MgCl_2_ and 500 μM acetosyringone) and mixed at a 1:1 ratio with p19 silencing suppressor. Tobacco (*N. benthamiana*) leaves were infiltrated using a needleless syringe and incubated under continuous white light for 3 days. Leaves were harvested, frozen in liquid nitrogen, and homogenized. sRNAs were extracted as described above, and mature miRNAs were detected using Xeno-R detection kit (Xenohelix, South Korea).

### Statistical analysis

Statistical analyses and figures were generated using GraphPad Prism 8.0.2 software. Quantitative data represent mean ± standard deviation (SD) from three biological replicates. Statistical tests and significance levels are described in each figure legend.

## Supplementary Material

Web_Material_uhaf312

## Data Availability

The Illumina sRNA sequencing data will be deposited at ENA Accession PRJEB101758 Additional data supporting this article are available in the article and in its online supplementary material. Predicted miRNA candidates in mung bean are available at https://doi.org/10.6084/m9.figshare.29411522.
