# Supplementary material for: Identification of specific miRNAs in early-stage mung bean (Vigna radiata) using DNA/AgNCs sensors and miRNAtome analysis
Source: Hortic Res. 2025 Nov 13;13(2):uhaf312. doi: 10.1093/hr/uhaf312 (PMC12946678; doi:10.1093/hr/uhaf312)
Supplement: Web_Material_uhaf312 [file web_material_uhaf312.zip › MungBean_Horticultural research_supple_revised_Final.pdf]

Figure S1

A

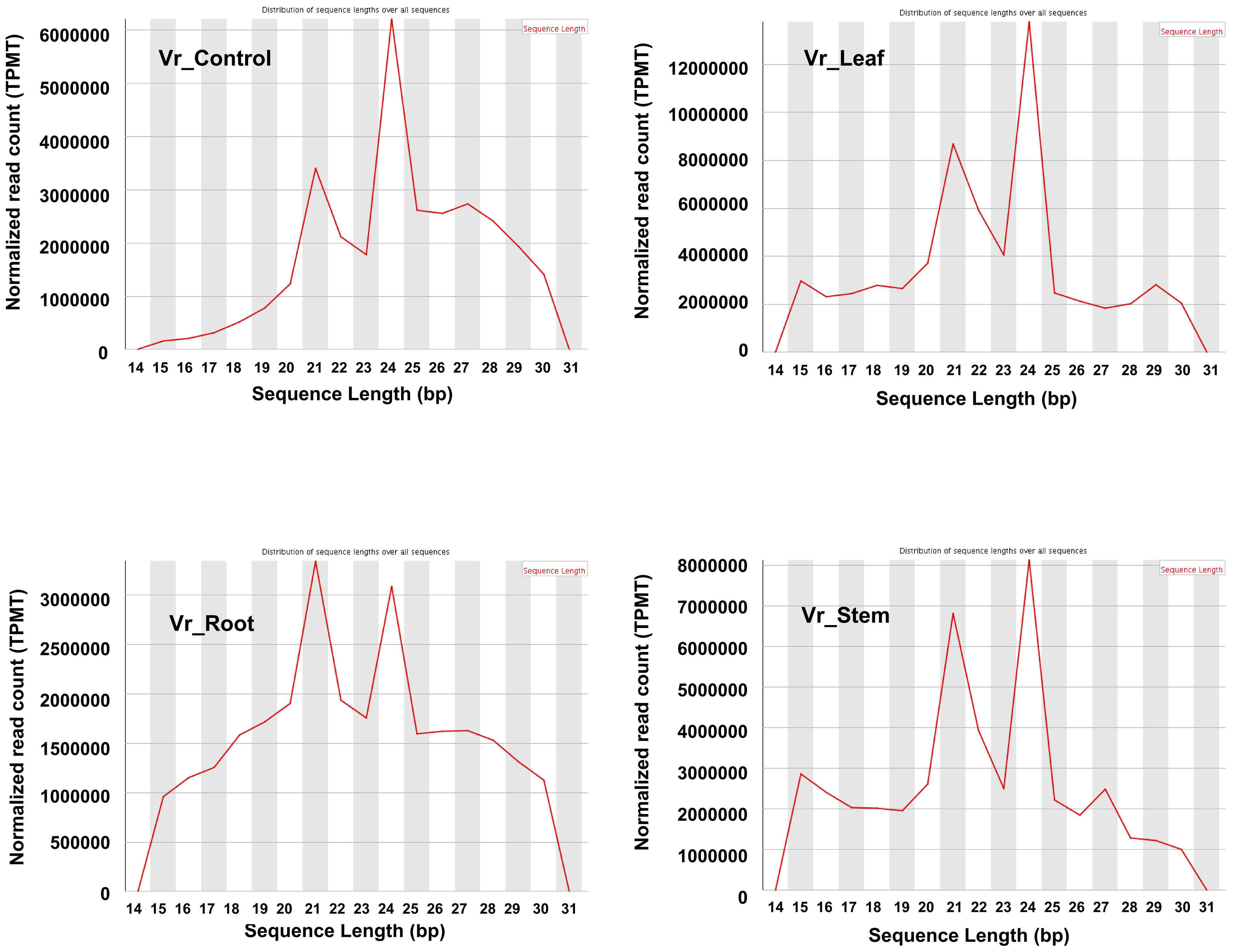

B

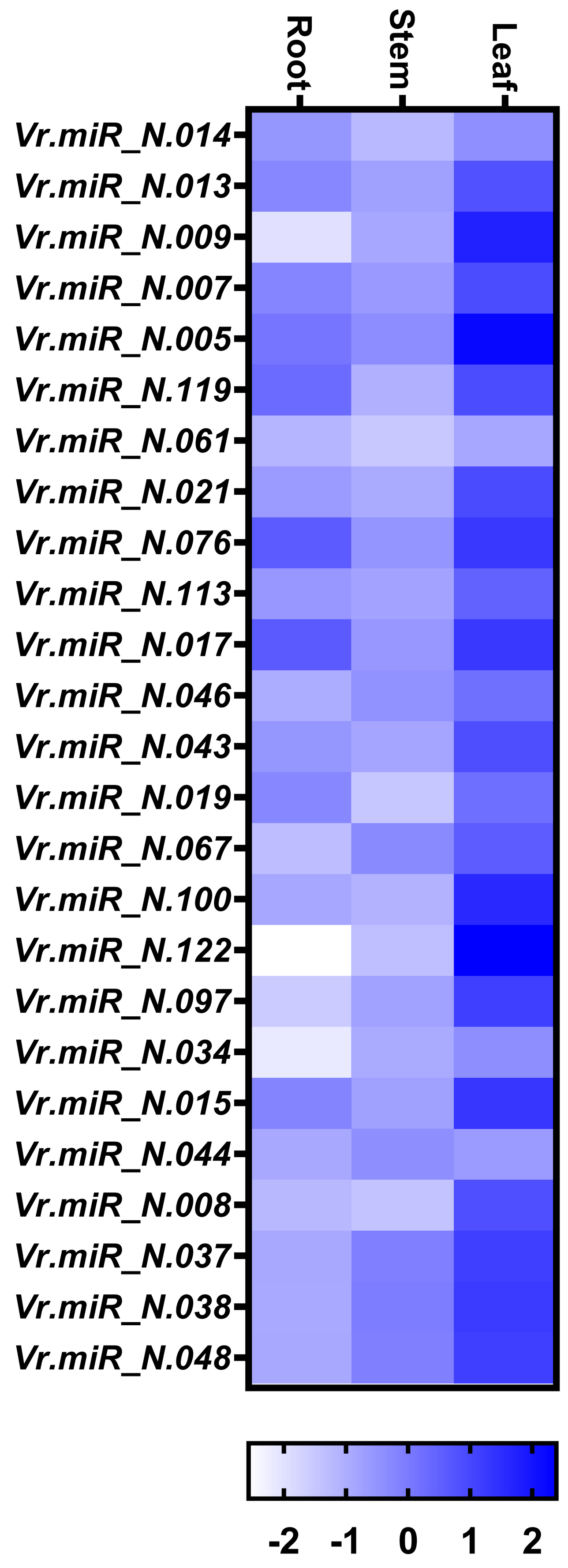

C

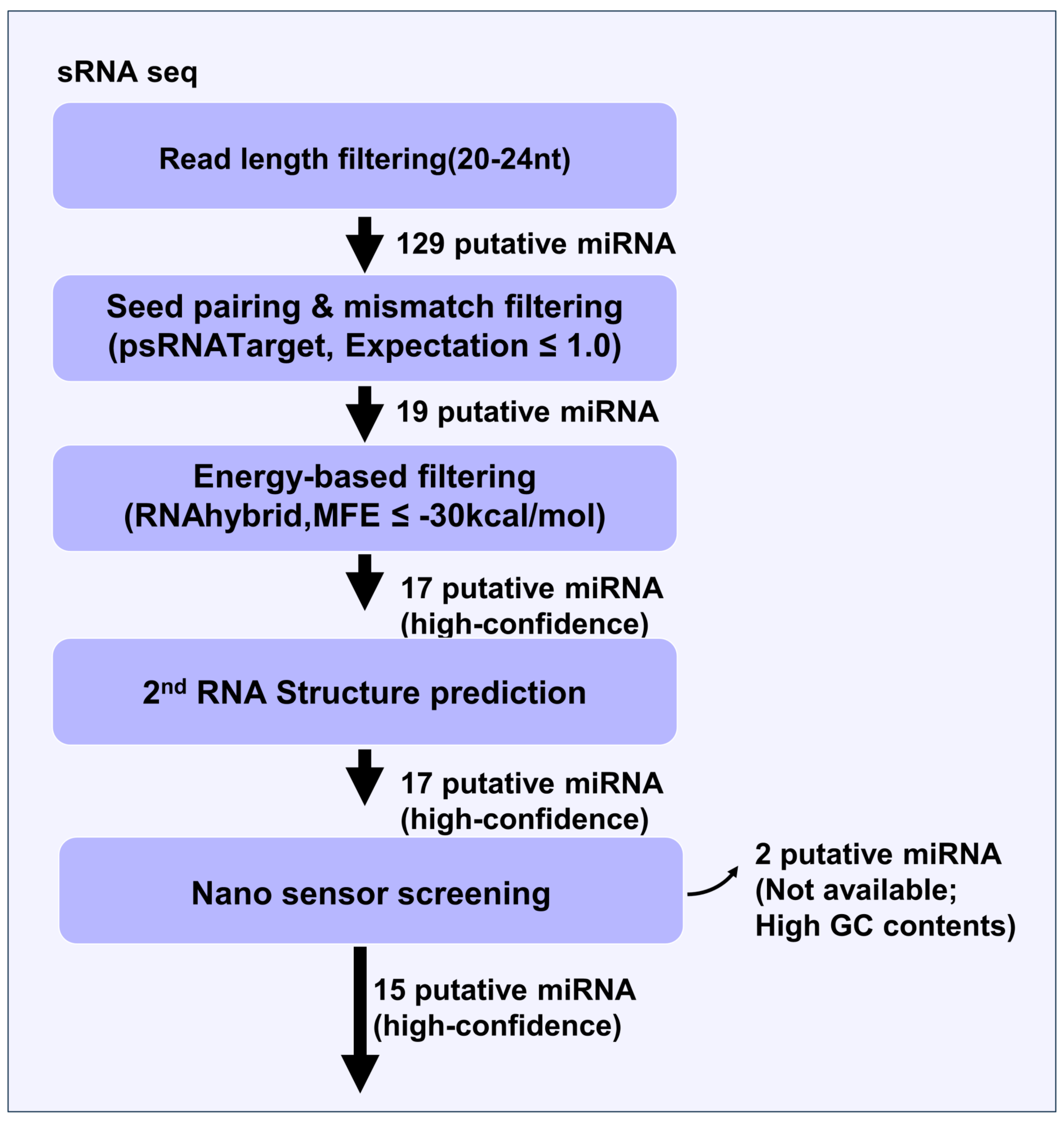

D

Mungbean specific miRNA	miRNA sequence	miRNA length (nt)	Target gene in mungbean	Annotate in A.thaliana gene	mde	Mfe (kcal/mol)
Vr.miR_N.007	GGUACAUGGACGAAUCGAACAUA	23	Vradi07g14160.1	uncharacterized gene	-46.999996	-37.6
Vr.miR_N.016	UGAAAUCGUCCAAUUACAUUUU	22	Vradi07g11170.1	IAMH2(AT4G37560)	-37.400005	-34.1
Vr.miR_N.022	UUUACGUGUCAUUCUGUGAUUG	22	Vradi0215s00340.1	PME1(AT4G12390)	-41.099998	-37.9
Vr.miR_N.026	AGAAAUGUAUGAAUUUAGUCCUU	23	Vradi08g10540.1	uncharacterized gene	-39.200005	-31.2
Vr.miR_N.027	GACACGUAGAUGAUAUUCAAAUA	23	Vradi01g01080.1	uncharacterized gene	-40.599998	-33.2
			Vradi05g09690.1	FLZ(AT4G17670)	-40.599998	-31.8
Vr.miR_N.041	UGAGAGAGGGACUAAAACCA	20	Vradi01g01000.1	UMAMIT41(AT3G28050)	-42.599998	-37.9
Vr.miR_N.045	UUGACAACAUUUGAACAUCAUAU	23	Vradi0215s00340.1	PME1(AT4G12390)	-38.899998	-35.4
Vr.miR_N.054	UGAAAUCGUCCAACUACAUUGUG	23	Vradi07g11170.1	IAMH2(AT4G37560)	-44.400002	-35.7
Vr.miR_N.068	UUUGGACCAAUCACACAAUGAUA	23	Vradi0007s01990.1	GPDHP(AT5G40610)	-43.299999	-35.3
			Vradi0360s00020.1	MSMO1-2(AT4G22756)	-43.299999	-35.3
			Vradi07g01300.1	FNR2(AT1G20020)	-43.299999	-35.3
Vr.miR_N.075	GAAAUCGUCCAAUUACAUUUU	21	Vradi07g11170.1	IAMH2(AT4G37560)	-35.200001	-32
Vr.miR_N.079	UCAUUUGGACCAAUCACACAAUG	23	Vradi07g01300.1	FNR2(AT1G20020)	-44.400002	-36
Vr.miR_N.087	GAAUGGACCUAGGAAACGACCCA	23	Vradi11g10830.1	TRX2(AT5G39950)	-51.199997	-37.6
Vr.miR_N.092	UUUGACAACAUUUGAACAUUUA	22	Vradi0215s00340.1	PME1(AT4G12390)	-34.699997	-31.2
Vr.miR_N.106	AAGAGAUCCAAAAGACGCAAGUU	23	Vradi08g04170.1	F-box/kelch-repeat protein-AT3G23880 like	-44.700005	-36.4
Vr.miR_N.110	AUUUACGUGUCAUUCUGUGAUUG	23	Vradi0215s00340.1	PME1(AT4G12390)	-42.400002	-39
Vr.miR_N.114	CCUGGCUCCCUGUAUGCCAU	20	Vradi0269s00010.1	ARF17(AT1G77850)	-47.499996	-45.7
			Vradi0279s00040.1	ARF16(AT4G30080)	-47.499996	-45.7
			Vradi02g05890.1	ARF16(AT4G30080)	-47.499996	-44.8
			Vradi04g02860.1	ARF16(AT4G30080)	-47.499996	-44.8
			Vradi05g10740.1	ARF17(AT1G77850)	-47.499996	-45.7
			Vradi08g16880.1	ARF16(AT4G30080)	-47.499996	-44.8
Vr.miR_N.129	UUUGACAACAUUUGAACAUCGUC	23	Vradi0215s00340.1	PME1(AT4G12390)	-41.499996	-37.2

Figure S2

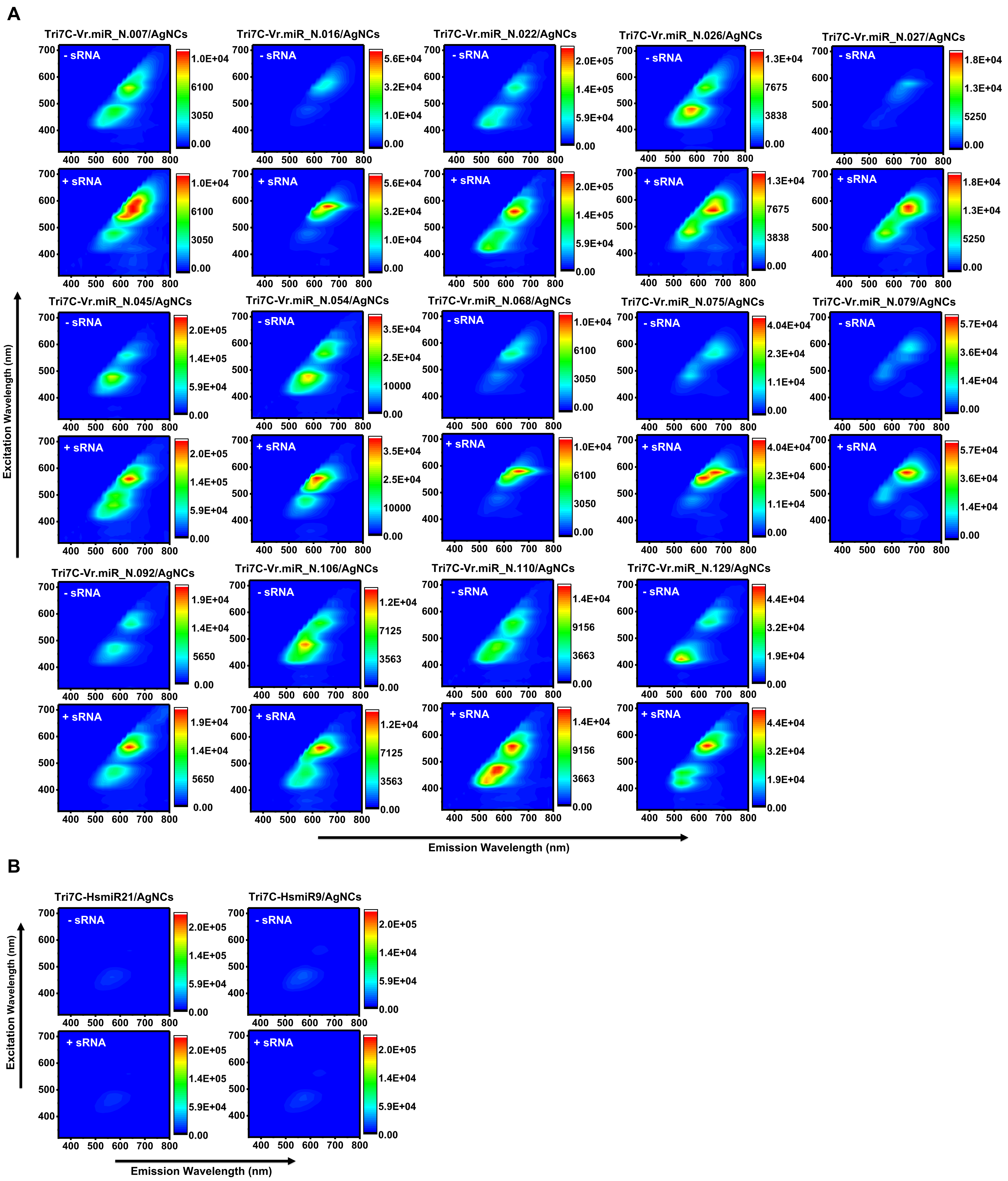
A
Tri7C-Vr.miR_N.007/AgNCs
Tri7C-Vr.miR_N.016/AgNCs
Tri7C-Vr.miR_N.022/AgNCs
Tri7C-Vr.miR_N.026/AgNCs
Tri7C-Vr.miR_N.027/AgNCs
Tri7C-Vr.miR_N.045/AgNCs
Tri7C-Vr.miR_N.054/AgNCs
Tri7C-Vr.miR_N.068/AgNCs
Tri7C-Vr.miR_N.075/AgNCs
Tri7C-Vr.miR_N.079/AgNCs
Tri7C-Vr.miR_N.092/AgNCs
Tri7C-Vr.miR_N.106/AgNCs
Tri7C-Vr.miR_N.110/AgNCs
Tri7C-Vr.miR_N.129/AgNCs
- sRNA
+ sRNA
Excitation Wavelength (nm)
Emission Wavelength (nm)
B
Tri7C-HsmiR21/AgNCs
Tri7C-HsmiR9/AgNCs
- sRNA
+ sRNA
Excitation Wavelength (nm)
Emission Wavelength (nm)

Figure S3

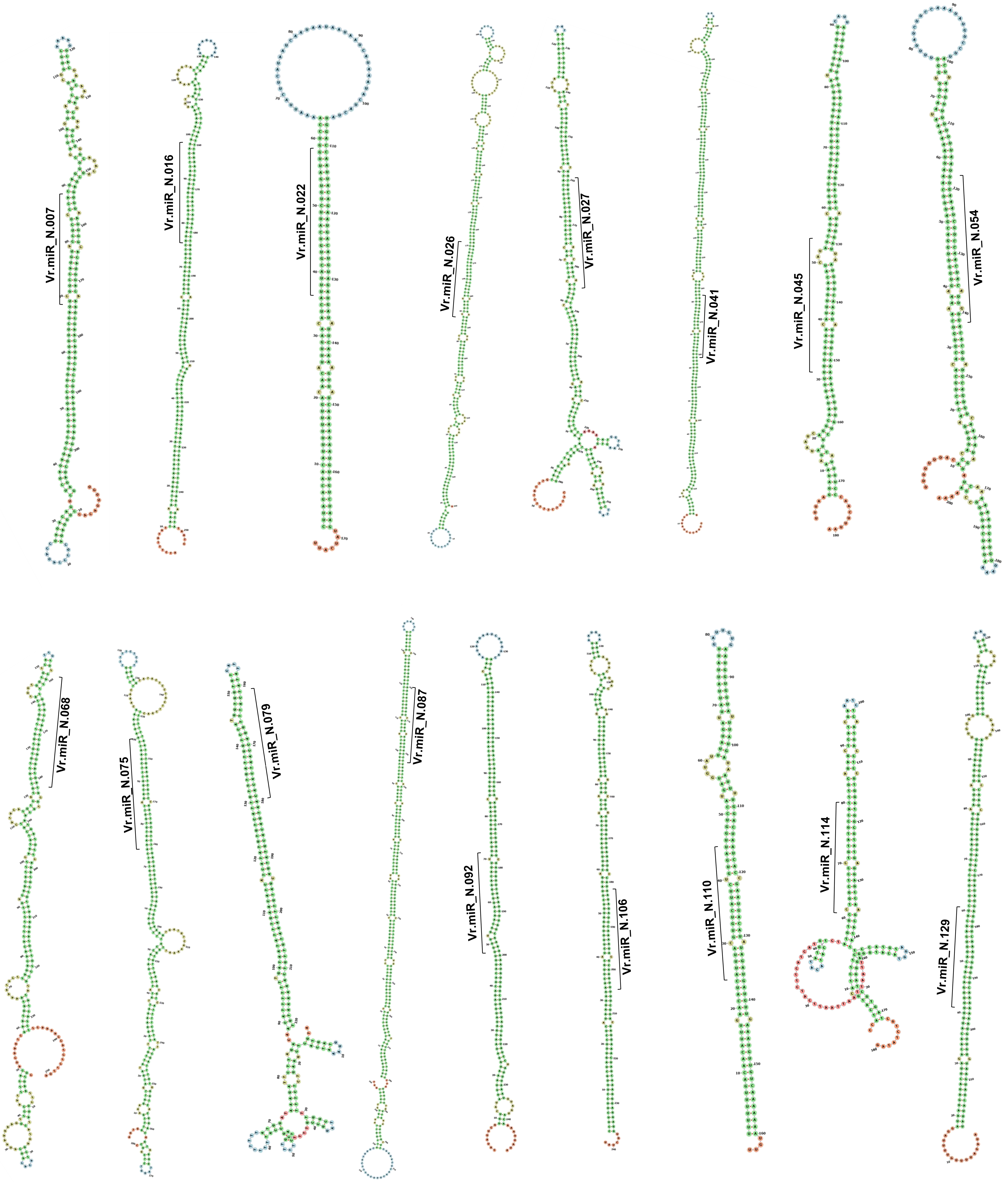
Vr.miR_N.007
Vr.miR_N.016
Vr.miR_N.022
Vr.miR_N.026
Vr.miR_N.027
Vr.miR_N.041
Vr.miR_N.045
Vr.miR_N.054
Vr.miR_N.068
Vr.miR_N.075
Vr.miR_N.079
Vr.miR_N.087
Vr.miR_N.092
Vr.miR_N.106
Vr.miR_N.110
Vr.miR_N.114
Vr.miR_N.129

Figure S4

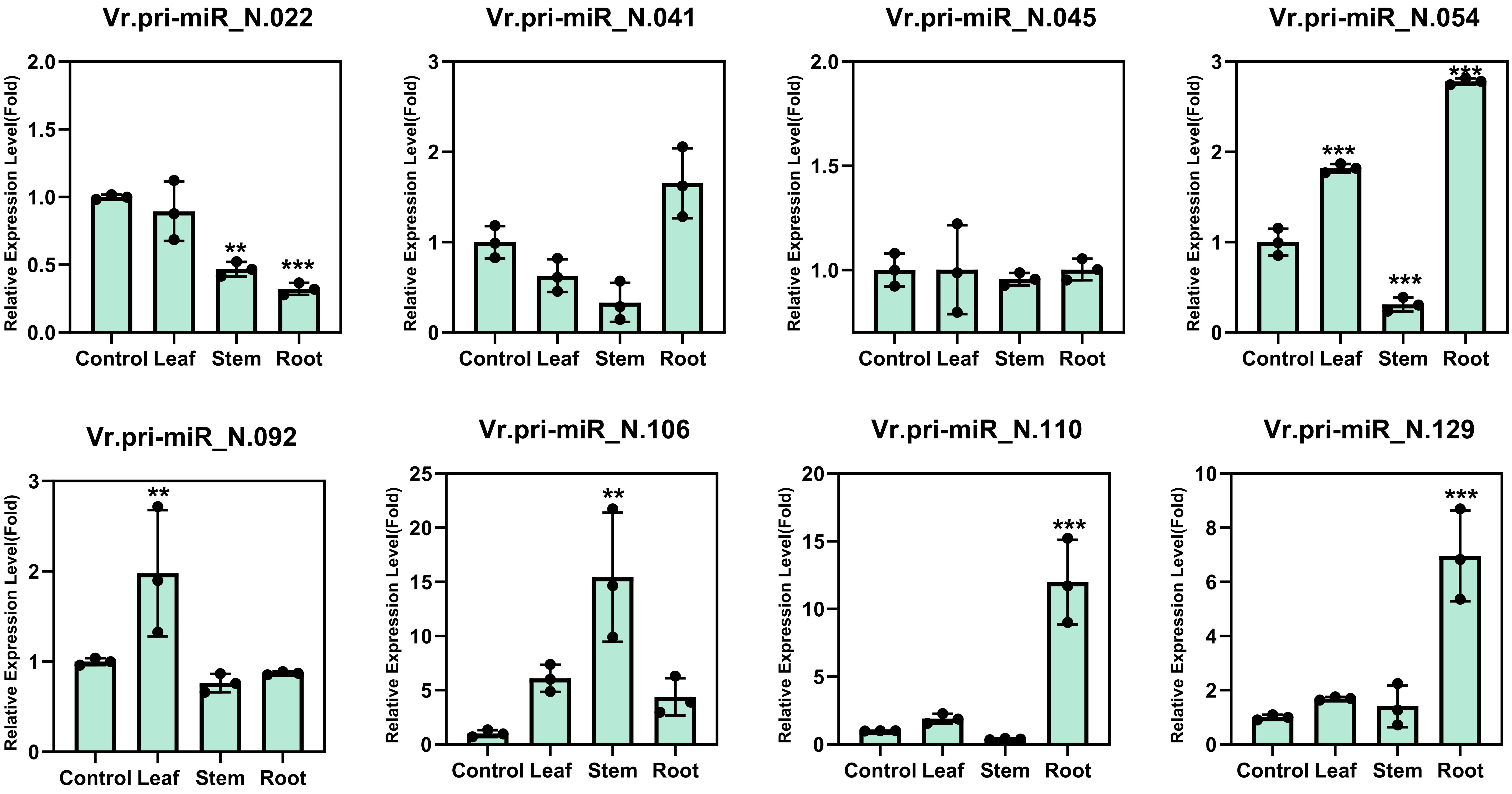
Vr.pri-miR_N.022
Vr.pri-miR_N.041
Vr.pri-miR_N.045
Vr.pri-miR_N.054
Vr.pri-miR_N.092
Vr.pri-miR_N.106
Vr.pri-miR_N.110
Vr.pri-miR_N.129
Relative Expression Level(Fold)
Control
Leaf
Stem
Root
**
***

Figure S5

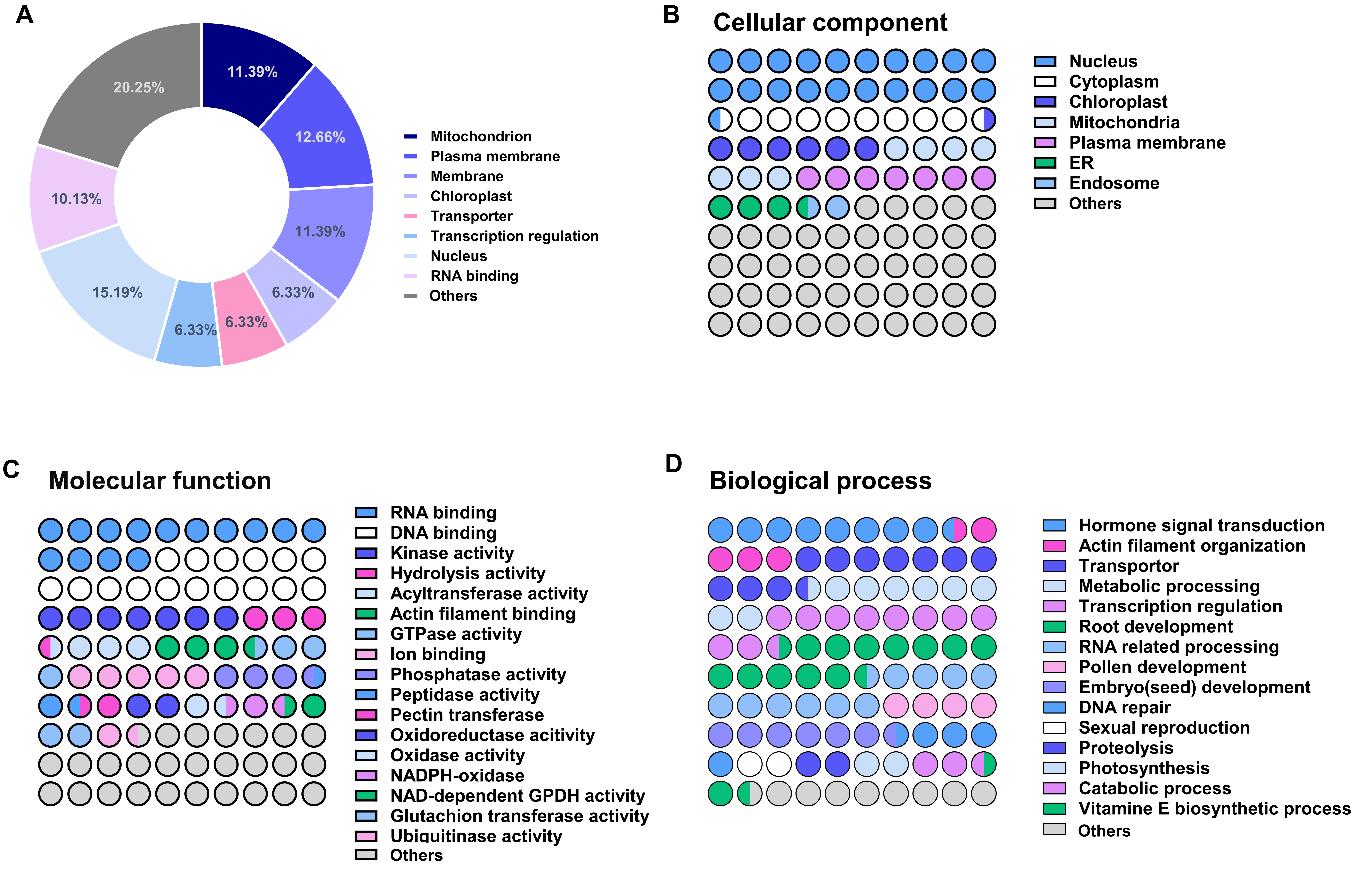
A
11.39%
12.66%
11.39%
6.33%
6.33%
6.33%
15.19%
10.13%
20.25%
Mitochondrion
Plasma membrane
Membrane
Chloroplast
Transporter
Transcription regulation
Nucleus
RNA binding
Others
B
Cellular component
Nucleus
Cytoplasm
Chloroplast
Mitochondria
Plasma membrane
ER
Endosome
Others
C
Molecular function
RNA binding
DNA binding
Kinase activity
Hydrolysis activity
Acyltransferase activity
Actin filament binding
GTPase activity
Ion binding
Phosphatase activity
Peptidase activity
Pectin transferase
Oxidoreductase acitivity
Oxidase activity
NADPH-oxidase
NAD-dependent GPDH activity
Glutachion transferase activity
Ubiquitinase activity
Others
D
Biological process
Hormone signal transduction
Actin filament organization
Transportor
Metabolic processing
Transcription regulation
Root development
RNA related processing
Pollen development
Embryo(seed) development
DNA repair
Sexual reproduction
Proteolysis
Photosynthesis
Catabolic process
Vitamine E biosynthetic process
Others
