# Supplementary material for: Identification of specific miRNAs in early-stage mung bean (Vigna radiata) using DNA/AgNCs sensors and miRNAtome analysis
Source: Hortic Res. 2025 Nov 13;13(2):uhaf312. doi: 10.1093/hr/uhaf312 (PMC12946678; doi:10.1093/hr/uhaf312)
Supplement: Web_Material_uhaf312 [file web_material_uhaf312.zip › Oh et al._Horticultural Research_Supporting data_revised.docx]

**Supporting Data**

**Identification of specific miRNAs in early-stage mung bean (*Vigna radiata*) using DNA/AgNCs sensors and miRNAtome analysis**

Young Kyoung Oh^1^, Hari Chandana Yadavalli^1^, Christian Møller^1^, Moon Young Ryu^2^, Seok Keun Cho^2^, Bora Lee^2^, Mikyung Chang^1^, Mi Young Byun^2^, Jong Hum Kim^3,4^, Hyun Ju Jung^1^* and Seong Wook Yang^1, 2^*

^1^Department of Systems Biology, Institute of Life Science and Biotechnology, Yonsei University, Seoul

03722, Korea.

^2^Xenohelix Research Institute, BT Centre 305, 56 Songdogwahakro Yeonsugu, Incheon 21984, Korea

^3^Department of Life Science, Pohang University of Science and Technology (POSTECH), 77 Cheongam-

Ro, Nam-Gu, Pohang, Gyeongbuk, 37673, Korea

^4^Institute for Convergence Research and Education in Advanced Technology, Yonsei University, 03722,

Korea

* Corresponding authors

Hyun Ju Jung [dokgu82@yonsei.ac.kr](mailto:dokgu82@yonsei.ac.kr)

Seong Wook Yang [yangsw@yonsei.ac.kr](mailto:yangsw@yonsei.ac.kr)


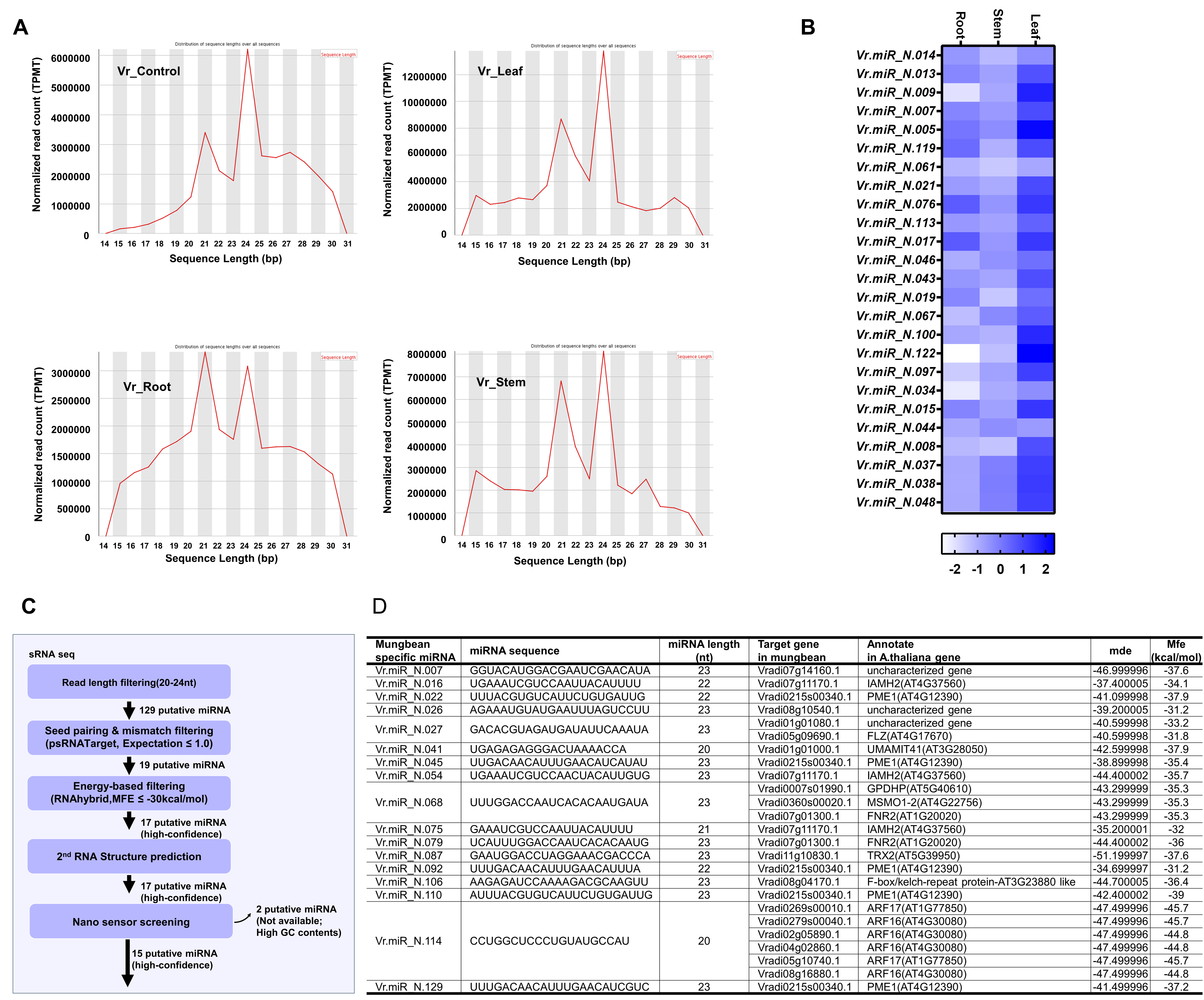


**Figure S1. Mung bean sRNA landscape and novel miRNA candidates**

Small RNA profiling and novel miRNA prediction in mung bean **(A)** sRNA profiles in mung bean were generated through sRNA sequencing analysis. Size distribution of sRNAs, across five-day-old whole seedling (grown for four days at dark conditions followed by light treatment for one day), leaf, stem, and root of mung bean. **(B)** the expression profile of the top 25 highly expressed novel mung bean miRNA across various mung bean tissues. **(C)** Schematic workflow of novel miRNA identification in mung bean, illustrating the computational pipeline and filtering steps. Key software tools and criteria applied for prediction and selection are indicated. **(D)** Summary of novel mung bean miRNAs identified through the computational pipeline illustrated in (C), along with their predicted target genes.


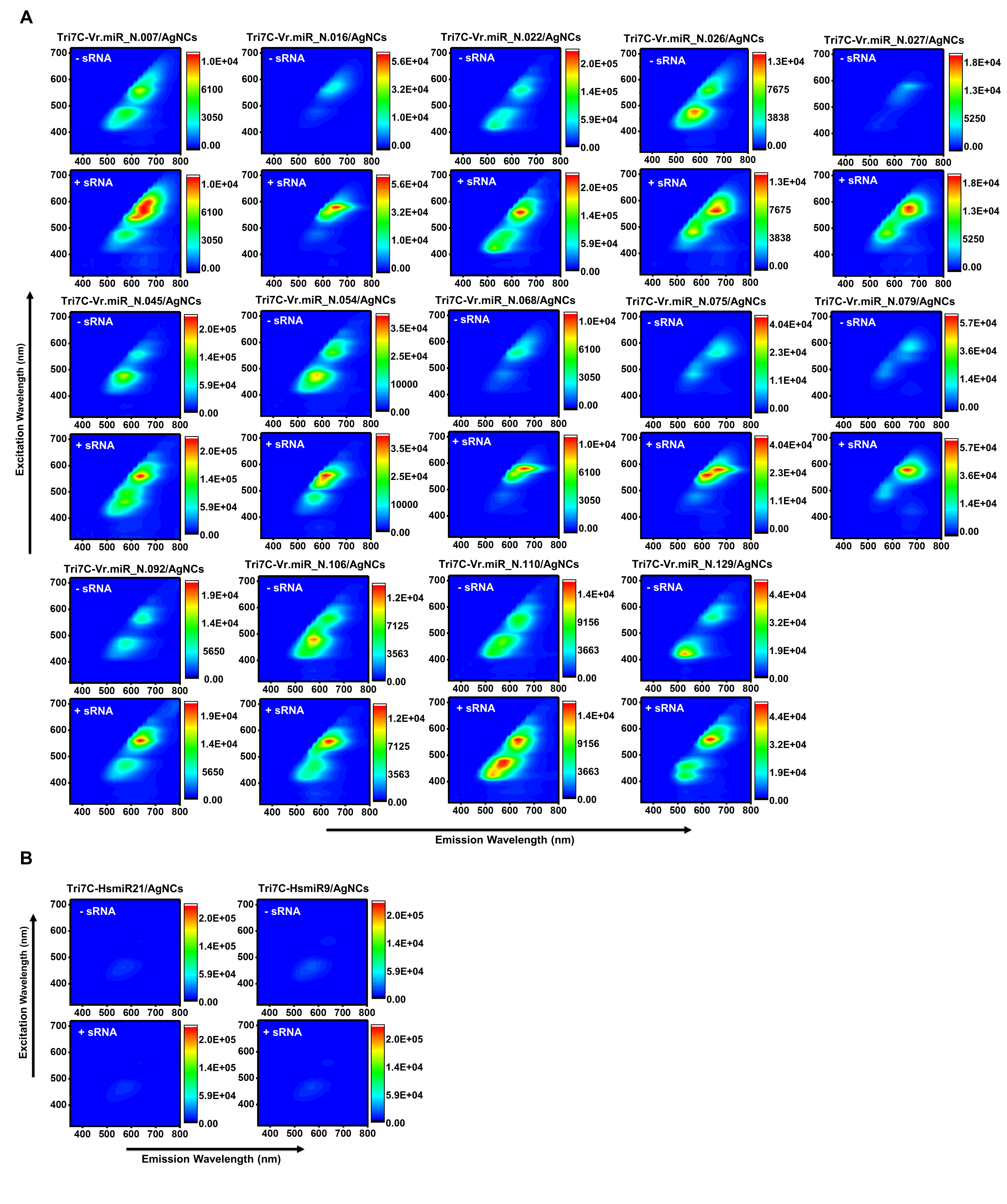


**Figure S2. Fluorescence emission of Tri7C-Vr.miR DNA/AgNCs sensors in the presence or absence of mung bean sRNA**

Fluorescence emission plot of 14 mung bean-specific Tri7C-Vr.miR DNA/AgNCs sensors in the presence or absence of mung bean sRNA. Tri7C-HsmiR21/AgNCs and -HsmiR9/AgNCs (mammalian miRNA detection sensor) were used as negative controls.

**
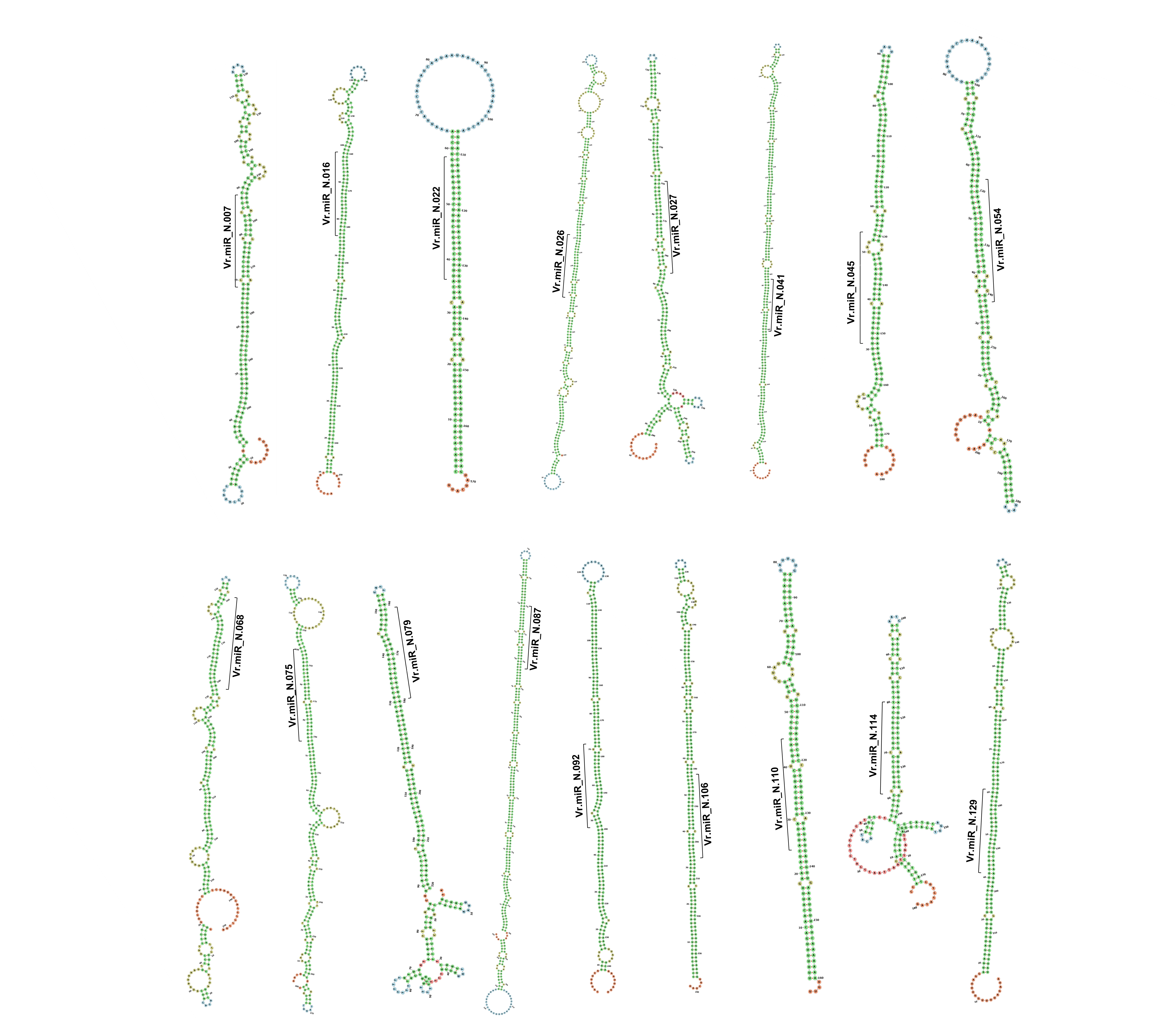
**

**Figure S3. Pri-miRNA prediction for mung bean-specific miRNAs**

Predicted pri-miRNA and secondary structures of selected 17 mung bean-specific miRNAs (Vr.miR_N.007, Vr.miR_N.016, Vr.miR_N.022, Vr.miR_N.026, Vr.miR_N.027, Vr.miR_N.041, Vr.miR_N.045, Vr.miR_N.054, Vr.miR_N.068, Vr.miR_N.075, Vr.miR_N.079, Vr.miR_N.087, Vr.miR_N.092, Vr.miR_N.106, Vr.miR_N.110, Vr.miR_N.114, Vr.miR_N.129). These 17 miRNAs were selected based on high predicted interaction scores between the miRNAs and their target mRNAs.


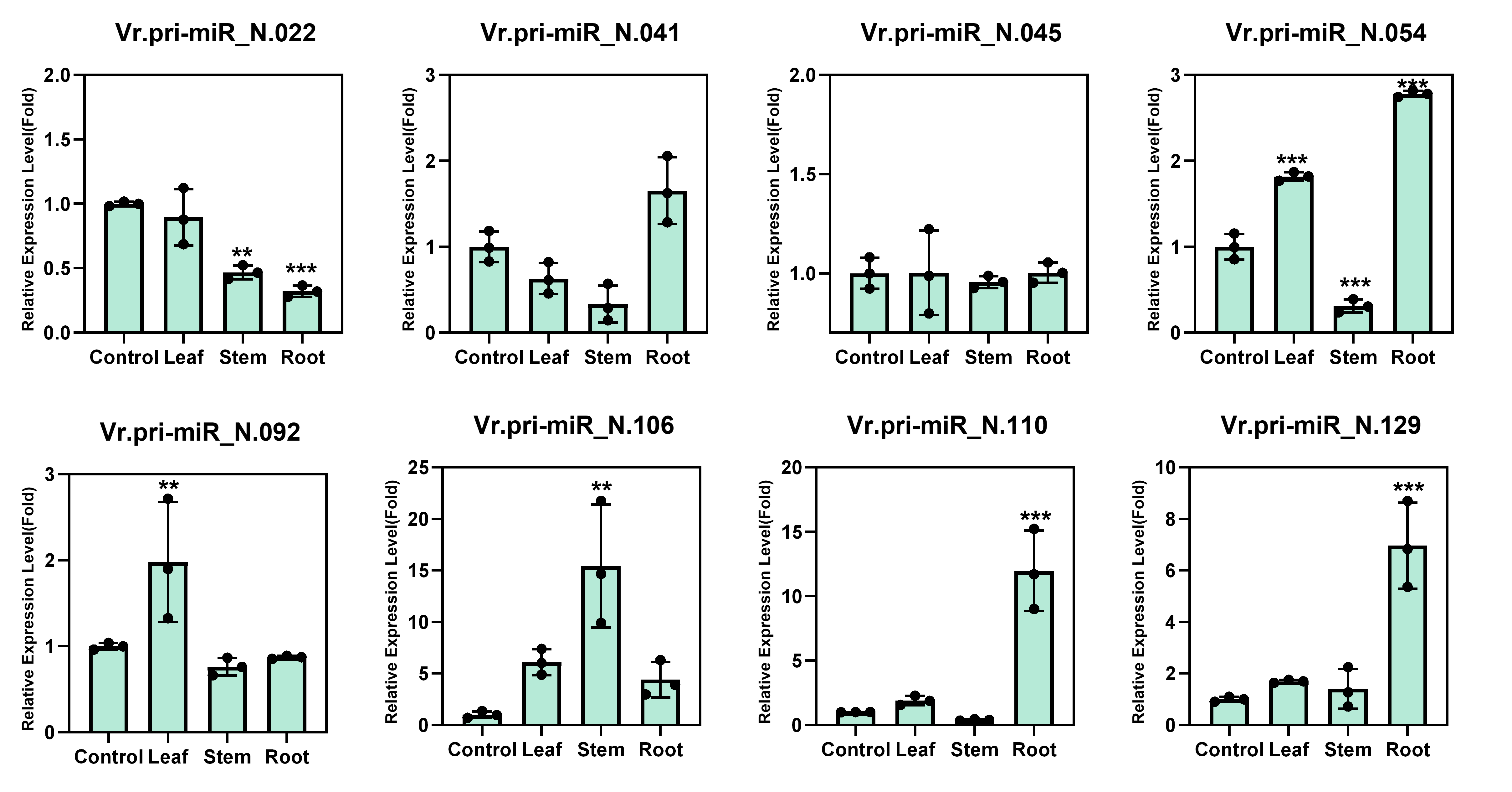


**Figure S4. Validation of** **mung bean-specific pri-miRNA expressions**

The predicted pri-miRNA structures of selected eight mung bean specific miRNAs. The expressions of the pri-miRNAs in mung bean were validated using qRT-PCR. Eight mung bean-specific miRNAs were selected based on high predicted interaction scores between the miRNAs and their target mRNAs. The primer used for validation is described in Table S4. The data were analyzed using, n=3 biological replicates. Emission signals were measured from three independent replicates and are presented as mean ± standard deviation (SD). Statistical significance was determined using a two-tailed unpaired Student’s *t*-test. *P* < 0.05 was considered statistically significant. Asterisks indicate significant differences compared to the control condition: *P* < 0.05 (**), P < 0.01 (**), P < 0.001 (****).


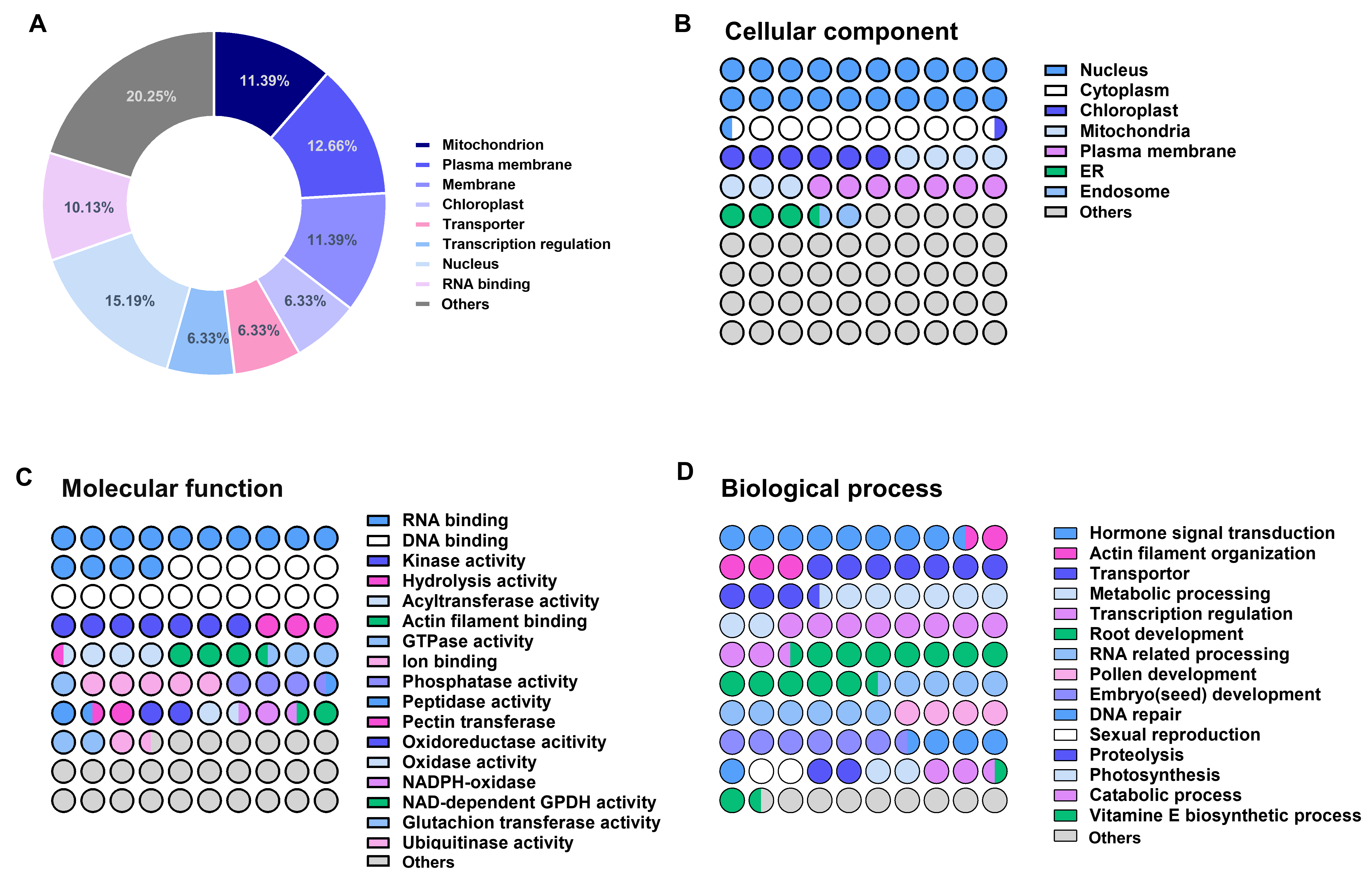


**Figure S5. Gene Ontology analysis of mung bean-specific miRNA target genes**

Gene Ontology (GO) analysis was conducted on target genes of mung bean-specific miRNAs. **(A)** Overall GO profile providing a comprehensive view of target gene classification, **(B-D)** Distribution of target genes within the (B) Cellular component, (C) Molecular function, and (D) Biological process, showing the roles of these target genes in various biological pathways.

**Table S1. Sequence of Tri7C-miR probes for miRNA detection**

| **Primer** | **Primer sequence (5’ 🡪 3’)** |
| --- | --- |
| Triplex_Vr.miR_N.007 | AAAAAAAAAATTTATTTTTTTTTTCCCCCCCTTT TTT TTT T TATGTTCGATTCGTCCATGTACC |
| Triplex_Vr.miR_N.016 | AAAAAAAAAATTTATTTTTTTTTTCCCCCCCTTT TTT TTT T AAAATGTAATTGGACGATTTCA |
| Triplex_Vr.miR_N.022 | AAAAAAAAAATTTATTTTTTTTTTCCCCCCCTTT TTT TTT T CAATCACAGAATGACACGTAAA |
| Triplex_Vr.miR_N.026 | AAAAAAAAAATTTATTTTTTTTTTCCCCCCCTTT TTT TTT T AAGGACTAAATTCATACATTTCT |
| Triplex_Vr.miR_N.027 | AAAAAAAAAATTTATTTTTTTTTTCCCCCCCTTT TTT TTT T TATTTGAATATCATCTACGTGTC |
| Triplex_Vr.miR_N.041 | AAAAAAAAAATTTATTTTTTTTTTCCCCCCCTTT TTT TTT T TGGTTTTAGTCCCTCTCTCA |
| Triplex_Vr.miR_N.045 | AAAAAAAAAATTTATTTTTTTTTTCCCCCCCTTT TTT TTT T ATATGATGTTCAAATGTTGTCAA |
| Triplex_Vr.miR_N.054 | AAAAAAAAAATTTATTTTTTTTTTCCCCCCCTTT TTT TTT T CACAATGTAGTTGGACGATTTCA |
| Triplex_Vr.miR_N.068 | AAAAAAAAAATTTATTTTTTTTTTCCCCCCCTTT TTT TTT T TATCATTGTGTGATTGGTCCAAA |
| Triplex_Vr.miR_N.075 | AAAAAAAAAATTTATTTTTTTTTTCCCCCCCTTT TTT TTT T AAAATGTAATTGGACGATTTC |
| Triplex_Vr.miR_N.079 | AAAAAAAAAATTTATTTTTTTTTTCCCCCCCTTT TTT TTT T CATTGTGTGATTGGTCCAAATGA |
| Triplex_Vr.miR_N.092 | AAAAAAAAAATTTATTTTTTTTTTCCCCCCCTTT TTT TTT T TAAATGTTCAAATGTTGTCAAA |
| Triplex_Vr.miR_N.106 | AAAAAAAAAATTTATTTTTTTTTTCCCCCCCTTT TTT TTT T AACTTGCGTCTTTTGGATCTCTT |
| Triplex_Vr.miR_N.110 | AAAAAAAAAATTTATTTTTTTTTTCCCCCCCTTT TTT TTT T CAATCACAGAATGACACGTAAAT |
| Triplex_Vr.miR_N.129 | AAAAAAAAAATTTATTTTTTTTTTCCCCCCCTTT TTT TTT T GACGATGTTCAAATGTTGTCAAA |
| Tri7C-hs-miR21 | AAAAAAAAAATTTATTTTTTTTTTCCCCCCCTTT TTT TTT T CAACATCAGTCTGATAAGCTA |
| Tri7C-hs-miR9 | AAAAAAAAAATTTATTTTTTTTTTCCCCCCCTTT TTT TTT T CATACAGCTAGATAACCAAAGA |
| Tri7C-ath-miR172a | AAAAAAAAAATTTATTTTTTTTTTCCCCCCCTTT TTT TTT T AGAATCTTGATGATGCTGCAT |
| Tri7C-ath-miR157a | AAAAAAAAAATTTATTTTTTTTTTCCCCCCCTTT TTT TTT T GACAGAAGATAGAGAGCAC |

**Table S2. Sequence of qPCR primer for mung bean-specific miRNA target gene detection**

| **Primer** | **Primer sequence (5’ 🡪 3’)** |
| --- | --- |
| Vr01g01000_qPCR_FW | CTGCCAGTTTTGGTCCTTCTTG |
| Vr01g01000_qPCR_RV | AAGCACAGTGGTTGCAACAG |
| Vr07g11170_qPCR_FW | TTGCATACTCGACCTTTGGC |
| Vr07g11170_qPCR_RV | TGCAGCTTCCTTTGCAATCC |
| Vr08g04170_qPCR_FW | TGTTTTGGGTTCCTGCAACG |
| Vr08g04170_qPCR_RV | CCATCCGAAACCAGCATTCTTC |
| Vr0215s00340_qPCR_FW | TGGAACACATGTGCAGCAAC |
| Vr0215s00340_qPCR_RV | TTTTGGCCAACGTTGCTGTC |
| Vr0007s01990_qPCR_FW | AAGTTGCCGATGCTTATGCC |
| Vr0007s01990_qPCR_RV | ACCTCATGCACCTCTTTTGC |
| Vr0360s00020_qPCR_FW | ATTTTGCGCCAGCTAGAAGC |
| Vr0360s00020_qPCR_RV | TAGTCATGGAATGCTGCTCCTC |
| Vr07g01300_qPCR_FW | ATGTTGGCAACTGGAACTGG |
| Vr07g01300_qPCR_RV | AAGAACAGCCATGCCAAACC |
| Vr05g09690_qPCR_FW | TGCAGAAGGAACAACACCAC |
| Vr05g09690_qPCR_RV | TTGCGTCCCAAACCATGATG |

**Table S3. Sequence of qPCR primer for mung bean-specific pri-miRNA detection**

| **Primer** | **Primer sequence (5’ 🡪 3’)** |
| --- | --- |
| Vr_premiR022_FW | CTTAGACAACATTTTTTTGACAACATTT |
| Vr_premiR022_RV | CCTTTTAGTTATATACACTCCCCTT |
| Vr_premiR041_FW | CCAACCCTAGTTTTTAAGTAAAGGT |
| Vr_premiR041_RV | GATAAAACAACAATGTTTGACGC |
| Vr_premiR045_FW | GTGATATCACATTCCCAACTT |
| Vr_premiR045_RV | CATTTGATGAAAGTAATTTATTGCTG |
| Vr_premiR054_FW | CGTCAAAATATCATTGTCCTTG |
| Vr_premiR054_RV | TTGTGTAGTTTGTGATTGGTC |
| Vr_premiR092_FW | GAAACCAATAGTATACTAAAAGAACG |
| Vr_premiR092_RV | CCACTTAAACAATTTTTTTTTATATC |
| Vr_premiR106_FW | CGA TGA CAT GAG AAA TAG GTA AGG |
| Vr_premiR106_RV | GAT GGA TTA TCT TCC ATT TGA ACC G |
| Vr_premiR110_FW | GGGATAATGATACTTAGAGAACATT |
| Vr_premiR110_RV | GACAACATTTTTTTGACAACATTTC |
| Vr_premiR129_FW | GTGGTATCATAAATGATAAAATTACTAACT |
| Vr_premiR129_RV | CTGAAATTATTTTGACAGGTAACAG |

**Table S4. Sequence of top 25 highly expressed mung bean-specific miRNA**

| **Identified miRNAs** | **LM (nt) ^2^** | **miRNA sequences** | **Strand** | **Chromosome** | **Start** | **End** |
| --- | --- | --- | --- | --- | --- | --- |
| Vr.miR_N.014 | 21 | TCGTGAAGGTTGAGTGCTTCC | + | Chr1 | 7933001 | 7933022 |
| Vr.miR_N.013 | 21 | TCATGAAAGTTGAGTGCTTTC | + | Chr1 | 7937740 | 7937761 |
| Vr.miR_N.009 | 21 | GTCATGAAGGTTGAGTGCTTT | + | Chr1 | 7935771 | 7935792 |
| Vr.miR_N.007 | 23 | GGTACATGGACGAATCGAACATA | - | Chr1 | 3054380 | 3054403 |
| Vr.miR_N.005 | 23 | GAAAACTCTGGTTTTAGTCCCTT | + | Chr1 | 3310421 | 3310444 |
| Vr.miR_N.119 | 23 | TTAGAATTCATGATATATAGGAT | - | Chr8 | 37259636 | 37259659 |
| Vr.miR_N.061 | 23 | TGAGATGACGTCGTTAAGAAGGT | - | Chr3 | 12949959 | 12949982 |
| Vr.miR_N.021 | 22 | TTGAAGGACTAAATTGTACCCT | - | Chr1 | 12785883 | 12785905 |
| Vr.miR_N.076 | 23 | GAAGTTTTTGAACATGTGGCACG | - | Chr5 | 1372857 | 1372880 |
| Vr.miR_N.113 | 24 | CAGTTTTCTAAAGTTGAAGGACTA | - | Chr8 | 27058996 | 27059020 |
| Vr.miR_N.017 | 24 | TGAAGTTTTTGAACATGTGGCACG | + | Chr1 | 24935868 | 24935892 |
| Vr.miR_N.046 | 23 | TTGTAGGACCGAGAGACTAACTT | + | Chr11 | 12712981 | 12713004 |
| Vr.miR_N.043 | 23 | TGTTTTCTAAAGTTGAAGGACTA | + | Chr11 | 1823128 | 1823151 |
| Vr.miR_N.019 | 23 | TTAGAATTCATAATATACAAGGG | - | Chr1 | 14896364 | 14896387 |
| Vr.miR_N.067 | 23 | TTACAGGGACTGTTGACTACCCC | + | Chr4 | 5973124 | 5973147 |
| Vr.miR_N.100 | 20 | GGTAGAAAGGACTAAAACCA | + | Chr7 | 45627926 | 45627946 |
| Vr.miR_N.122 | 20 | AATTCTTCGGATATGCTGTA | - | Chr9 | 7976385 | 7976405 |
| Vr.miR_N.097 | 22 | CGTAAGAACTCCATGGTTAAGC | + | Chr7 | 40661503 | 40661525 |
| Vr.miR_N.034 | 20 | AGAACTCCACAGAATGCTGG | + | Chr11 | 3481372 | 3481392 |
| Vr.miR_N.015 | 23 | TGAAAACTCTGGTTTTAGTCCTT | + | Chr1 | 20745046 | 20745069 |
| Vr.miR_N.044 | 23 | TTACCGAGGGCTTTCTGACCGTC | + | Chr11 | 4507831 | 4507854 |
| Vr.miR_N.008 | 23 | GTCAGACATATAGACGTCGGTCT | + | Chr1 | 6965363 | 6965386 |
| Vr.miR_N.037 | 24 | GAATTTTTTAAAGTTGAAGGACTA | + | Chr11 | 2570713 | 2570737 |
| Vr.miR_N.038 | 23 | GCGAGAACTAAACTGAACATCCG | + | Chr11 | 12291126 | 12291149 |
| Vr.miR_N.048 | 23 | AATTTTTTAAAGTTGAAGGACTA | + | Chr2 | 23612038 | 23612061 |
